# Supplementary material for: Therapeutic radiographers’ delivery of health behaviour change advice to those living with and beyond cancer: a qualitative study
Source: BMJ Open. 2020 Aug 11;10(8):e039909. doi: 10.1136/bmjopen-2020-039909 (PMC7422652; doi:10.1136/bmjopen-2020-039909)
Supplement: Supplementary data [file bmjopen-2020-039909supp001.pdf]

Supplementary material 1: Interview topic guide

Topic	Question	Prompt
**Introduction**	Brief introductions; get verbal consent to tape-record the interview; remind the aims of study, check length of interview/audio-recorded.	
**Health promotion beliefs**	What are your views on the role of health behaviours in those with a diagnosis of cancer?<br><br>Do you think delivering advice on improving health behaviours is part of your role?	Breast/prostate/colorectal/head & neck cancers<br><br>Healthy eating/ exercise/ alcohol/ smoking / weight management
**Provision of advice on healthy behaviours**	Can you tell me about your experiences of providing health behaviour advice to cancer patients?<br><br>Are there some health behaviours you recommend more than others?<br><br>Why do you think that is?<br><br>Are there some patient groups that you provide health behaviour advice to more than others?<br><br>Why do you think that is?	Healthy eating/ exercise/ alcohol/ smoking / weight management
**Barriers to health advice delivery**	On the survey we asked you about a number of barriers limiting you in providing health behaviour advice to your patients. You mentioned X Y Z<br><br>Can you tell me more about these barriers?<br><br>Can you think of any other barriers within your role and work environment stopping you from giving lifestyle and health promotion advice to patients?	Why do you think that is?
**Addressing barriers**	What support do you think would be most helpful in enabling therapeutic radiographers to deliver health behaviour advice to patients?	
**Training preferences**	How helpful do you think an online training course specifically for therapeutic radiographers on delivering health behaviour advice to cancer patients would be?<br><br>How helpful do you think a face to face training course specifically for therapeutic radiographers on delivering health behaviour advice to cancer patients would be?<br><br>If you were provided with a training course (online or face to face) what topics would you like delivered?	Why do you think this is?
**Finish**	Thank you for your time. Do you have anything else you would like to add?	
