# Supplementary material for: Therapeutic radiographers’ delivery of health behaviour change advice to those living with and beyond cancer: a qualitative study
Source: BMJ Open. 2020 Aug 11;10(8):e039909. doi: 10.1136/bmjopen-2020-039909 (PMC7422652; doi:10.1136/bmjopen-2020-039909)
Supplement: Author's manuscript [file bmjopen-2020-039909.draft_revisions.pdf]

BMJ Open is committed to open peer review. As part of this commitment we make the peer review history of every article we publish publicly available.

When an article is published we post the peer reviewers' comments and the authors' responses online. We also post the versions of the paper that were used during peer review. These are the versions that the peer review comments apply to.

The versions of the paper that follow are the versions that were submitted during the peer review process. They are not the versions of record or the final published versions. They should not be cited or distributed as the published version of this manuscript.

BMJ Open is an open access journal and the full, final, typeset and author-corrected version of record of the manuscript is available on our site with no access controls, subscription charges or pay-per-view fees (http://bmjopen.bmj.com).

If you have any questions on BMJ Open's open peer review process please email info.bmjopen@bmj.com

BMJ Open

Therapeutic radiographers' practices and facilitators for delivering health behaviour change advice to those living with and beyond cancer: a qualitative study.

Journal:	*BMJ Open*
Manuscript ID	bmjopen-2020-039909
Article Type:	Original research
Date Submitted by the Author:	29-Apr-2020
Complete List of Authors:	Pallin, Nickola; University College London, Behavioural Science and Health; London South Bank University, Department of Allied Health Sciences<br>Beeken, Rebecca; University College London, Behavioural Science and Health; University of Leeds Leeds Institute of Health Sciences<br>Pritchard-Jones, Kathy; London Cancer<br>Charlesworth, Laura; University of Lincoln<br>Woznitza, Nicholas; Homerton University Hospital NHS Foundation Trust, Radiology; Canterbury Christ Church University, Canterbury Christ Church University<br>Fisher, Abigail; University College London, Behavioural Science and Health
Keywords:	RADIOTHERAPY, MEDICAL EDUCATION & TRAINING, Adult oncology < ONCOLOGY, QUALITATIVE RESEARCH

SCHOLARONE™
Manuscripts

I, the Submitting Author has the right to grant and does grant on behalf of all authors of the Work (as defined in the below author licence), an exclusive licence and/or a non-exclusive licence for contributions from authors who are: i) UK Crown employees; ii) where BMJ has agreed a CC-BY licence shall apply, and/or iii) in accordance with the terms applicable for US Federal Government officers or employees acting as part of their official duties; on a worldwide, perpetual, irrevocable, royalty-free basis to BMJ Publishing Group Ltd ("BMJ") its licensees and where the relevant Journal is co-owned by BMJ to the co-owners of the Journal, to publish the Work in this journal and any other BMJ products and to exploit all rights, as set out in our licence.

The Submitting Author accepts and understands that any supply made under these terms is made by BMJ to the Submitting Author unless you are acting as an employee on behalf of your employer or a postgraduate student of an affiliated institution which is paying any applicable article publishing charge ("APC") for Open Access articles. Where the Submitting Author wishes to make the Work available on an Open Access basis (and intends to pay the relevant APC), the terms of reuse of such Open Access shall be governed by a Creative Commons licence – details of these licences and which Creative Commons licence will apply to this Work are set out in our licence referred to above.

Other than as permitted in any relevant BMJ Author's Self Archiving Policies, I confirm this Work has not been accepted for publication elsewhere, is not being considered for publication elsewhere and does not duplicate material already published. I confirm all authors consent to publication of this Work and authorise the granting of this licence.

For peer review only

Title: Therapeutic radiographers' practices and facilitators for delivering health behaviour change advice to those living with and beyond cancer: a qualitative study.

Authors

Pallin N D [1, 2] Beeken R J [1,3] Pritchard Jones K [4] Charlesworth L[5] Woznitza N[6] Fisher A[1]

1. Department of Behavioural Science and Health, University College London, London, UK
2. Department of Allied Health Sciences, London South Bank University, London, UK.
3. Leeds Institute of Health Sciences, University of Leeds, Leeds, UK
4. London Cancer, London, UK
5. College of Social Science, University of Lincoln, Lincolnshire, UK.
6. Radiology Department, Homerton University Hospital, London, UK.

Nickola Pallin email: pallinn@lsbu.ac.uk

Dr Rebecca Beeken email: R.Beeken@leeds.ac.uk

Prof Kathy Pritchard Jones: k.pritchard-jones@ucl.ac.uk

Laura Charlesworth: LCharlesworth@lincoln.ac.uk

Dr Nick Woznitza: Nicholas.woznitza@nhs.net

Dr Abi Fisher email: Abigail.fisher@ucl.ac.uk

Present address of corresponding author: Nickola Pallin

School of Health and Social Care

Department of Allied Health Sciences

London South Bank University

103 Borough Road

London

SE1 0AA

Tel: 0207815674

Email: pallinn@lsbu.ac.uk

Note: This work was undertaken in the Department of Behavioural Science and Health, University College London, London, UK. The lead author was based there as a pre-doctoral research fellow.

For peer review only - http://bmjopen.bmj.com/site/about/guidelines.xhtml

Abstract

Objectives Therapeutic radiographers (TRs) are well placed to deliver health behaviour change advice to those living with and beyond cancer (LWBC). However, there is limited research on the opinions of TRs around delivering such advice to those LWBC. This study aimed to explore TRs' practices and facilitators in delivering advice on physical activity, healthy eating, alcohol intake, smoking and weight management

Setting and Participants Fifteen UK-based TRs took part in a telephone interview using a semi-structured interview guide. Data was analysed using the framework analysis method.

Results Emergent themes highlighted that TRs are mainly aware of the benefits of healthy behaviours in managing radiotherapy treatment related side effects, with advice provision lowest for healthy eating and physical activity. Participants identified themselves as well placed to deliver advice on improving behaviours to those LWBC, however reported a lack of knowledge as a limiting factor to doing so. The TRs reported training and knowledge as key facilitators to the delivery of advice, with a preference for online training.

Conclusions There is a need for education resources, clear referral pathways and in particular training for TRs on delivering physical activity and healthy eating advice to those LWBC.

For peer review only - http://bmjopen.bmj.com/site/about/guidelines.xhtml

Strengths and limitations of this study

- This study provides insight in therapeutic radiographers' views on all key modifiable health behaviours for those living with and beyond cancer.
- The participants worked in different radiotherapy departments, offering insight into the practices among therapeutic radiographers in the delivery of healthy behaviour advice from a wide range of hospitals.
- Whilst the sample size was small data saturation was reached.
- Participants might be more interested in the role of health behaviours in cancer survivorship, which might bias the responses towards a positive view on the role of therapeutic radiographers' in delivering advice within their role.

Keywords: Therapeutic Radiographer, cancer, health behaviours, healthy eating, physical activity, lifestyle, behaviour change

peer review only

Introduction

It is estimated that 40% of cancer cases are linked to unhealthy behaviours [1]. Based on evidence from systematic literature reviews and meta analyses, the World Cancer Research Fund (WCRF) recommend that individuals are physically active, limit consumption of energy dense foods, salty foods, red meat and avoid processed meat, eat more plant foods, maintain a healthy weight, limit alcoholic drinks and avoid tobacco to reduce their risk of cancer [2]. Those living with and beyond cancer (LWBC) are also advised to follow these guidelines, due to increasing evidence that healthy behaviours may improve physical and psychosocial outcomes after a cancer diagnosis [2- 8].

Despite the potential benefits of healthy behaviours, few people LWBC are meeting the WCRF recommended health behaviour recommendations [9, 10]. Those LWBC report one key reason for not adopting healthier lifestyle behaviours is lack of advice and support from their healthcare team [11]. Healthcare professionals (HCPs) are well placed to bring about positive health behaviour changes among cancer patients [12]. A trial of brief advice among breast cancer survivors showed that a simple physical activity recommendation from a HCP doubled the percentage meeting national exercise guidelines [12]. Despite this, research to date among both HCPs and those LWBC consistently shows that few oncology HCPs offer guidance to oncology patients on healthy lifestyle behaviours [13 – 21].

Reported barriers among HCPs in providing health behaviour advice for those LWBC, include believing that giving advice was not part of their role, lack of time with patients, lack of referral programmes, lack of resources such as education leaflets for those LWBC, and lack of knowledge regarding guidelines and research findings [16 – 21]. A recent qualitative study with 21 oncology HCPs identified that advice on health behaviours provided to those LWBC focused on general health and controlling side effects, with few HCPs advising on health behaviours in the context of improving survival outcomes [20]. Whilst these studies provide useful insight into the practices and barriers among oncology HCPs the participants within these studies were primarily oncologists and nurses and focused on the provision of physical activity and weight management advice. There is limited research on the opinions of therapeutic radiographers (TRs) in delivering advice on health behaviours to those LWBC. Despite at least 50% of cancer patients receiving radiotherapy as part of their cancer treatment [22].

For peer review only - http://bmjopen.bmj.com/site/about/guidelines.xhtml

TRs are the only health professionals qualified to deliver radiotherapy and play a central role in supporting cancer patients [23]. In the UK, the College of Radiographers recognise the importance of TRs in providing health behaviour advice to improve patient outcomes [24]. TRs are also seen as an integral part of the health force in driving improvements in wellbeing as outlined in the 2017 publication of "AHPs into Action, using Allied Health Professionals to transform health, care and wellbeing" which states that radiographers are key to implementing a preventative healthcare approach and that their expertise should be used to design and deliver health interventions [25]. TRs are ideally placed to deliver health behaviour advice, particularly through Making Every Contact Count (MECC) [26,27.] MECC is a strategy whereby health professionals use every appropriate opportunity and interaction with patients to promote healthy behaviours and signpost to relevant healthcare services using an 'Ask, Advise, Act' framework [27]. MECC fits extremely well within the TRs' role in which patient education is a key part of radiotherapy practice with TRs providing care to the same patient every day, often over a number of weeks [23]. TRs therefore have the potential to make significant contributions in supporting positive health behaviour changes among those LWBC.

However, despite these opportunities one survey in the UK among 102 TRs identified that TRs rarely advise patients on the key modifiable health behaviours including smoking, alcohol, healthy eating and exercise [15]. The findings also showed lack of knowledge and training as barriers among TRs in delivering advice on these topics [15]. Similarly, focus group interviews with 38 TRs identified that lack of knowledge and training were barriers to the provision of smoking cessation advice [28].

Challenges remain in translating behaviour change interventions into existing care pathways and practices in a way that is appropriate for use by health professionals [29]. Understanding TRs' practices, and what support they need in delivering advice on the topics of physical activity, healthy eating, alcohol intake, smoking and weight management could inform the development of interventions that will enable TRs' in delivering advice on improving health behaviours to those LWBC. Qualitative research is appropriate for exploring the beliefs, experiences and motivations of individuals on specific matters and allow for more information and clarification [30]. Limited qualitative data exists on TRs' practices and views on delivering advice on these health behaviour topics. This study therefore aimed to address this and through a qualitative methodology explore TRs' practices

For peer review only - http://bmjopen.bmj.com/site/about/guidelines.xhtml

in delivering health behaviour advice, in addition to exploring the facilitators in delivering such advice. Preferences regarding training on delivering this advice were also explored.

Methods

Participants and Recruitment

Participants were TRs working in the UK in a clinical role. They had provided their contact details on an online survey investigating TR's practices in delivering health behaviour advice agreeing to be invited for a follow up telephone interview. An email was sent with an information sheet explaining the research and inviting these TRs. Those who agreed to take part signed a consent form prior to the telephone interview. Ethical approval was granted by the University College London Research Ethics Committee, reference 12945/001.

Data collection

Semi-structured individual telephone interviews were carried out between April and May 2019 by a lecturer in therapeutic radiography with an MSc who had completed qualitative interviewing as part of their training (NP). The interviewer had no previous relationship with the study participants. The topic guide (supplementary material 1) was based on the guide used within a previous study [17] which explored oncology HCPs views on the provision of lifestyle advice to cancer patients. This guide was adapted for use among TRs, with additional questions added to assess preferences for training on delivering advice. The topic guide was piloted with two participants to check for comprehension of the questions. This data was included in the analysis because no substantial changes were required. The interviews lasted approximately 30 min (range: 20 – 40 min) and were audio-recorded, anonymised and transcribed verbatim. The transcripts were verified by NP against each recording to confirm accuracy. The aim was to carry out interviews until data saturation was reached. It was anticipated that ten participants would be required to reach data saturation because it was a homogeneous group [31]. After ten interviews were carried out, they were transcribed verbatim. Following familiarisation with the data NP generated the initial codes and it appeared that saturation was reached after ten interviews as no new codes occurred in the tenth interview [32]. A further five interviews were carried out to confirm this.

For peer review only - http://bmjopen.bmj.com/site/about/guidelines.xhtml

Patient and public involvement

Patient input was not used in the design of the research methods. However, the topic guide was piloted with TRs in the academic setting. Additionally, the topic guide was piloted with two participants and these were included in the analysis.

Analysis

The interview transcripts were analysed using the framework analysis method [33]. This method was chosen because it is an appropriate method for analysing homogenous data and semi-structured interview transcripts, it is also appropriate when using inductive qualitative analysis [33]. A random selection of transcripts (n = 3) were independently coded by AF to check for reliability. The researchers NP, AF and RJB met and agreed on a final coding list in an iterative process (AF and RJB are both experienced qualitative researchers and health psychologists). These agreed set of codes formed the analytical framework which was then applied to all of the transcripts and the data summarised in a matrix using Microsoft excel. Themes were generated by reviewing the matrix connecting the data between the participants and the codes. The completed consolidated criteria for reporting qualitative research checklist is available in the supplementary material 2 [34]. Themes are presented in the results with supporting quotes and the participants identifier (Table 1).

Table 1: Participant identifier and demographic characteristics

Participant Identifier	Demographic characteristics	
	**Gender**	**Professional Grade**
TR 1	Female	Band 6
TR 2	Female	Band 7
TR 3	Female	Band 7
TR 4	Female	Band 7
TR 5	Female	Band 7
TR 6	Female	Band 7
TR 7	Female	Band 7
TR 8	Male	Band 8
TR 9	Female	Band 7
TR 10	Female	Band 7
TR 11	Female	Band 6
TR 12	Female	Band 7
TR 13	Male	Band 5
TR 14	Female	Band 7
TR 15	Male	Band 8

TR: Therapeutic Radiographer

For peer review only - http://bmjopen.bmj.com/site/about/guidelines.xhtml

Results

Participants

The radiotherapy radiography workforce census in the UK only reports the workforce's professional grade, and no other demographics [35]. In the UK, TRs' level of professional skills and knowledge are categorised by agenda for change (AfC) professional band grades 5 to 8 [36]. Therefore, in this study, the participants gender and professional grade were collected; no other demographic information was collected (Table 1). Seventy-two TRs were invited to take part in an interview and 15 returned consent forms and completed the telephone interview. Fifteen interviews were conducted with 12 women and 3 men. The participants came from all regions of the UK, including England, Wales, Scotland and Northern Ireland.

Themes

Five main themes were identified: (1) TRs provide behaviour change advice to manage radiotherapy related side effects (2) TRs make judgements about when it is appropriate to deliver health behaviour advice (3) Knowledge and training are key facilitators in the delivery of health behaviour advice (4) TRs feel patients undergoing radiotherapy treatment seek guidance on health behaviours (5) TRs identify themselves as well placed to give health behaviour advice to patients.

TRs provide behaviour change advice to manage radiotherapy related side effects

Most respondents reported that they only provided advice on health behaviours that they believed would minimise radiotherapy related side effects. This meant smoking cessation and alcohol intake were the two health behaviours TRs mainly advised on.

"With head and neck patients, we give advice, particularly on smoking and drinking, obviously get worse side effects" (TR 6).

"The only thing we do generally say is about drinking plenty of fluids, avoiding alcohol. But that's more to do with prostate side effects, bladder reactions, and reducing gas" (TR 14).

For peer review only - http://bmjopen.bmj.com/site/about/guidelines.xhtml

"Radiographer are comfortable talking about alcohol when it comes to managing side effects" (TR 12).

No TRs reported advising patients on healthy eating. Some TRs mentioned advising patients on dietary intake but this is to patients who are at risk of losing weight, for side effect management and potential impact on accuracy of radiotherapy treatment delivery.

"Healthy eating, I don't tend to discuss too much. A lot of patients have difficulty eating and we are encouraging maintaining weight while on treatment" (TR 5).

"I'm not very sure if healthy eating is important. Any patients where we're treating, lower GI or pelvis, we would advise them to avoid very high-fibre foods, spicy foods, that might make them have very loose bowels, but other than that we say more or less keep on your same diet. We wouldn't generally discuss a healthy diet as a standard for all patients. No" (TR 15).

Some TRs mentioned they advise patients to be physically active, however this was only in the context of managing radiotherapy and cancer related fatigue.

"So exercise is one of my main ones that I focus on with all patients, particularly to help with their fatigue" (TR 9).

"Exercise, I say that's its quite beneficial to help with fatigue" (TR 12).

"I guess when we have patients come in, fatigue is one of the side effects, so we encourage our patients to remain active" (TR 15).

TRs make judgements about when it is appropriate to deliver health behaviour advice

TRs explained only discussing health behaviours, particularly smoking and alcohol with patients if there were evident indications of a problem. TRs also often reported making a judgment of whether appropriate to advise a patient on a particular health behaviour.

For peer review only - http://bmjopen.bmj.com/site/about/guidelines.xhtml

"So, quite often you can tell if a patient is a smoker, you can smell it, or you can tell by their skin" (TR 11).

"I tend to give advice when you make a judgement of when it's appropriate, an example might be if a patient smell of smoke" (TR 12).

"Had patients come in and will smell of alcohol and at that time I'll say to the patient that it can exacerbate side effects" (TR 15).

This meant TRs did not provide advice on health behaviours to every patient.

"But for those patients where it's not clearly going to benefit them to stop drinking, you would just mention it very briefly. Not every patient will have that information" (TR 5).

Knowledge and training are keys facilitator in the delivery of health behaviour advice

Delivery of advice matched by knowledge

The reported delivery of advice on health behaviours appeared to be matched by knowledge of the benefits among those LWBC.

One participant explained how he only appreciated the importance of PA in cancer survivorship after attending a talk and being made aware of the evidence.

"My experience of appreciating the role exercise was from attending a talk. I suppose it was really just highlighting in the studies the benefits obviously of a healthy lifestyle and introducing physical activity for patients on treatment" (TR 15).

Healthy eating was a topic the participants felt particularly unqualified to deliver advice on, and reported lack of knowledge as a barrier to the delivery of advice on healthy eating.

For peer review only - http://bmjopen.bmj.com/site/about/guidelines.xhtml

"It's a difficult one, diet, I think. It's more a knowledge thing. If you don't have the knowledge about what you can and can't say, you're just not going to approach the subject" (TR 12).

A need for continuous post graduate online training

All interviewed said they would welcome post graduate training on delivering health behaviour advice. The majority expressed a preference for online training to help overcome the barrier of limited time among TRs to attend training.

"Online, you're not having to take time out of clinical practice, online is more accessible" (TR 6)

Participants also mentioned that online training allows for yearly updates and continuous professional development (CPD).

"I think it'd be good (online training) because you can do it in your own time. Because I think that's sometimes the problem. You have this training once and then maybe it never gets brought up again. So it would be quite handy to have something small, every year, alongside all your other mandatory training" (TR 12).

Participants did acknowledge that face to face training allows for further questioning that's not possible with online training.

"I think one to one training, because you can ask questions that may not be covered within the online training" (TR 1).

To overcome of the barrier of not all staff being able attend face to face training participants suggested it would be useful to train some TRs through face to face methods that they could then cascade to other TRs within the radiotherapy department.

"Maybe some face-to-face with some staff, that they could cascade down, might be useful as well" (TR 4).

For peer review only - http://bmjopen.bmj.com/site/about/guidelines.xhtml

A need for training in the undergraduate setting

It was also suggested to incorporate training on delivering lifestyle advice into the undergraduate education program.

"Certainly, get it into the undergraduate course to start with, making them aware it is part of the role" (TR 9).

"It's still not something that I can say was primarily covered in the undergraduates' training about the benefits of healthy lifestyle, you know, there's no real formal education that I can see" (TR 15).

TRs reported knowledge of resources and referral pathways as facilitators in the delivery of advice

Participants also felt knowledge of how to refer patients onto further support would enable them to have conversations on improving health behaviours. With some TRs reporting that lack of knowledge of resources and referral pathways are barriers to initiating a conversation on behaviour change.

"There needs to be more information available to professionals of where exactly you can refer patients to, whether that be website, whether that be app" (TR 13).

"That's the only reason why they (therapeutic radiographers) don't want to open these conversations up, because they don't know where to go with it or how to refer on" (TR 9).

They also acknowledge that in the short time they have with patients if they had a resource then would be more inclined to advise.

"Having something on a piece of paper, education and having the resources, if you can do it in two minutes you should be able slip that in" (TR 2).

"You don't always have that information at hand, so if it was readily available, I think we'd give out a bit more (health behaviour information) if it was just the case of pointing them in the right direction that would be a quick and easy thing to do" (TR 3).

For peer review only - http://bmjopen.bmj.com/site/about/guidelines.xhtml

The benefit of incorporating patients' perspective into training

Participants also mentioned that getting patients' perspectives on receiving advice on improving health behaviours should be incorporated into training.

"I think that would be better coming from the patients themselves, rather than just feedback from what journals, and other literature says" (TR 7).

"If there'd even be patients that would be willing to maybe just even be involved with staff training" (TR 3).

TRs feel patients undergoing radiotherapy treatment seek guidance on health behaviours.

Many of the TRs also described that patients often ask them for guidance around health behaviour changes, particularly on diet and exercise. This shows that patients see TRs as credible sources of information on health behaviours.

"We are getting asked the question more and more about weight loss, healthy living, wanting to exercise more" (TR 4).

"It is quite a common thing to be asked at the end of treatment, not so much the smoking and alcohol, I have to say, but diet and exercise is certainly something that people commonly ask" (TR 3).

TRs identify themselves as well placed to give health behaviour advice to patients.

TRs acknowledged that they are a consistent healthcare member for patients undergoing radiotherapy and have many opportunities to deliver lifestyle advice. Therefore, TRs recognised that they are well placed to deliver health behaviour advice to patients.

"We're in a unique position because we do see the same patient day after day and you do kind of start to develop a relationship with them" (TR 10).

"I think we're well placed to help influence patients' behaviours and it' something we should be seen to encourage and report" (TR 7).

For peer review only - http://bmjopen.bmj.com/site/about/guidelines.xhtml

"We're in the best position where we see the patients, for a number of weeks every day to encourage any changes" (TR 8).

From the interviews it appeared that many patients undergoing radiotherapy, excluding those at risk of malnutrition or significant weight loss, are primarily reviewed and assessed by TRs. This highlights that TRs are in an ideal position to deliver advice on health behaviours, particularly when asked about nutrition advice delivery.

"They routinely see the specialist radiographer, for the breast patients. But they don't have a dietitian appointment" (TR 12).

"Prostate and breast are two tumour groups that are fully radiographer-led review and about 70% to 80% of our work load. They generally wouldn't be sent to a dietician" (TR 15).

"Only have a dietitian on board for the head and necks" (TR 9).

Discussion

TRs in this study saw themselves as well placed to deliver health behaviour advice, but also reported that they do not routinely provide advice to all patients. TRs were particularly unlikely to provide advice on healthy eating and physical activity, and were more likely to provide advice on those behaviours they believed would minimise radiotherapy or cancer related side effects. This is in line with previous research among TRs [15,28,37]. In one qualitative study a key facilitator reported among TRs in delivering smoking cessation support to patients was knowledge of the link between smoking and toxicity [28]. Another qualitative study that explored allied health professionals' views regarding the provision of dietary advice to patients, highlighted that TRs report giving dietary advice to help counteract the side effects of radiotherapy [37]. Additionally, in our study, if TRs did provide dietary advice, this tended to be general advice rather than cancer-specific advice on healthy eating.

In some studies, oncology HCPs have reported they do not self-identify as the right person to provide lifestyle

For peer review only - http://bmjopen.bmj.com/site/about/guidelines.xhtml

For peer review only

advice [17,20]. However, in this study TRs identified themselves as being well placed to deliver health behaviour advice and in a unique position as a consistent member of the multidisciplinary team providing care to patients. However, despite this, they do not feel qualified to deliver advice, particularly on the topic of healthy eating. In the UK, poor diet has the biggest impact on the NHS budget, greater than alcohol consumption, smoking and physical inactivity [38]. It has been noted that there are insufficient dietitians to provide dietary advice to all patients who may need dietary support [39]. In response to this all HCPs are being asked to implement a preventative healthcare approach within their role and the delivery of healthy eating advice is fundamental to this [23,24,40,41]. Key to achieving this is that TRs will have the skills, knowledge and behaviours to improve the health and wellbeing of individuals [24]. As with other oncology HCP groups [16,17,20,37] this study identifies the need for education and training among TRs in delivering health behaviour advice, particularly on healthy eating and physical activity. This training should also address when and how to refer to other support if necessary, as this was identified as a key facilitator in the delivery of advice on health behaviours, particularly when time is a barrier to the delivery of this advice [15,16,17].

All interviews demonstrated that TRs would welcome training on delivering health behaviour advice and recommended it as a key facilitator in delivering advice, in addition to incorporating it into the undergraduate setting. The need for post graduate training among TRs in delivering health advice has also recently been reported by Charlesworth et al. [28] in relation to the delivery of smoking cessation advice. Our findings from this study provide additional insight into TRs preferences on the type of training on delivering lifestyle advice to those LWBC, with TRs demonstrating a preference of online training in the post graduate setting. Amongst HCPs online education has been reported to be as effective as face-to-face education [42]. Additionally, the use of online learning enables HCPs to carry out training at time that fits in with clinical work [43, 44]. TRs in this study identified this benefit of online learning in overcoming the limited time available for TRs to undertake continuous professional development and additional training. Interestingly, TRs in this study mentioned having patient input in the training would be helpful. Whilst HCPs input is key to the development of interventions, patient members play key advocacy roles and their input can enhance the outcomes of interventions [45]. Patient input may also help overcome the reported barrier of fear of causing offence to a patient, which has been reported as a barrier amongst oncology HCPs in delivery of health behaviour advice [17].

For peer review only - http://bmjopen.bmj.com/site/about/guidelines.xhtml

Those LWBC wish to receive advice on health behaviours from their healthcare team [13,20,46]. This was made apparent in this study, whereby some TRs mentioned that healthy eating and exercise were the health behaviours patients ask for advice on more often, particularly towards the end of their treatment. This further highlights the importance of supporting TRs in delivering evidence-based health behaviour advice to meet patients' needs.

Of those interviewed in this study it appeared that those with breast, prostate and colorectal cancer are primarily reviewed and assessed by TRs. Therefore, it is the responsibility of TRs to deliver advice on improving health behaviours to these patients. This is also particularly important because the strongest evidence for the benefits of diet and exercise is currently in breast, prostate and colorectal cancer survivors [47]. These are also the most common cancers in the UK and radiotherapy plays a key role in managing these cancers [22, 48]. Therefore, with the right skills and knowledge, TRs could deliver advice on improving health behaviours. By supporting self-efficacy among patients towards the end of their treatment which very often is in the radiotherapy department can be empowering for patients. Among those with prostate cancer, implementing dietary changes brought psychological benefit, as a method of coping and regaining control over their diagnosis [46].

This study has strengths and limitations. This is the first qualitative study among TRs to explore the provision of advice on all key modifiable lifestyle behaviours for those LWBC as per recommendations [2]. Whilst the aim of qualitative research is not to generalise the findings, the participants worked in different radiotherapy departments and therefore provide insight into the practices among TRs in the delivery of healthy behaviour advice from a wide range of hospitals. Additionally, the participants worked in cancer centres in England, Wales, Scotland and Northern Ireland, providing insight into the practices across the UK. Whilst the sample size was small data saturation was reached, likely due the homogeneous sample of participants. Participants might be more interested in the role of health behaviours in cancer survivorship, which might bias the responses towards a positive view on this topic and the role of TRs in delivering advice within their role.

Conclusion

In conclusion, whilst the majority of TRs delivered some advice on health behaviours as part of their role, advice was mainly on smoking and alcohol intake. Most believed in the value of this advice in managing radiotherapy and cancer related side effects. Provision of advice was lowest for weight management, healthy eating and

For peer review only - http://bmjopen.bmj.com/site/about/guidelines.xhtml

physical activity. The findings show a clear need for training among TRs in delivering advice on improving health behaviours among those LWBC, with TRs reporting a preference for online training in the post graduate setting.

Declarations

Contributors: NDP had the original idea for the study and obtained the funding. All authors developed the study design and recruitment approach. NDP acquired the data. NDP, AF and RJB analysed the data. All authors approved the final version of submission.

Competing Interests: None

Funding: This research was funded by Cancer Research UK (ref no: C59769/A25965

Data sharing statement Data can be obtained by the corresponding author on reasonable request.

Ethics approval: The methodology for this study was approved by the Human Research Ethics committee of University College London (reference 12945/001).

Consent to participate: Informed consent was obtained from all individual participants included in the study.

Acknowledgments: The researchers are grateful to the health professionals who participated in the study.

view only

For peer review only - http://bmjopen.bmj.com/site/about/guidelines.xhtml

References

1. Brown KF, Rumgay H, Dunlop C, Ryan M, Quartly F, Cox A et al. The fraction of cancer attributable to modifiable risk factors in England, Wales, Scotland, Northern Ireland, and the United Kingdom in 2015. Br. J. Cancer. 2018;118(8):1130-41.
2. World Cancer Research Fund/American Institute for Cancer Research. Diet, Nutrition, Physical Activity and Cancer: a Global Perspective. Continuous Update Project Expert Report. 2018. [Internet] Available at: dietandcancerreport.org. Accessed 20 Jan 2020.
3. Thomas, R, Davies, N. Lifestyle during and after cancer treatment. Clin. Oncol. 2007;19(8): 616-627.
4. Chan DS, Vieira AR, Aune D, Bandera EV, Greenwood DC, McTiernan A et al. Body mass index and survival in women with breast cancer—systematic literature review and meta-analysis of 82 follow-up studies. Ann. Oncol. 2014 Oct 1;25(10):1901-14.
5. Speck, R.M., Courneya, K.S., Mâsse, L.C., Duval, S. & Schmitz, K.H. An update of controlled physical activity trials in cancer survivors: a systematic review and meta-analysis. J Cancer Surviv. 2010;4 (2):87-100
6. Pierce JP, Stefanick ML, Flatt SW, Natarajan L, Sternfeld B, Madlensky L et al. Greater survival after breast cancer in physically active women with high vegetable-fruit intake regardless of obesity. J. Clin. Oncol. 2007;25(17):2345–2351.
7. World Cancer Research Fund/American Institute for Cancer Research. Continuous Update Project Expert Report. 2018. Survivors of breast and other cancers. [Internet] Available at: https://www.wcrf.org/sites/default/files/Breast-cancer-survivors-report.pdf. Accessed: 16 Jan 2020. Accessed 2 April 2020.
8. Meyerhardt JA, Niedzwiecki D, Hollis D, Saltz LB, Hu FB, Mayer RJ et al. Association of dietary patterns with cancer recurrence and survival in patients with stage III colon cancer. Jama. 2007;298(7):754-64.
9. Tollosa DN, Tavener M, Hure A, James EL. Adherence to multiple health behaviours in cancer survivors: a systematic review and meta-analysis. J Cancer Surviv. 2019;13(3):327-43.
10. Pugh G, Hough R, Gravestock H, Fisher A. The health behaviour status of teenage and young adult cancer patients and survivors in the United Kingdom. Support. Care Cancer. 2020;28(2):767-77
11. Corbett T, Cheetham T, Müller AM, Slodkowska-Barabasz J, Wilde L, Krusche A et al. Exploring cancer survivors' views of health behaviour change: "Where do you start, where do you stop with everything?". Psycho-oncology. 2018;27(7):1816-24.

For peer review only - http://bmjopen.bmj.com/site/about/guidelines.xhtml

12. Jones, L.W., Courneya, K.S., Fairey, A.S., Mackey, J.R. Effects of an oncologist's recommendation to exercise on self-reported exercise behavior in newly diagnosed breast cancer survivors: a single-blind, randomized controlled trial. Ann. Behav. Med. 2004;28(2):105-13

13. Beeken RJ, Williams K, Wardle J, Croker H. "What about diet?" A qualitative study of cancer survivors' views on diet and cancer and their sources of information. Eur. J. Cancer Care. 2016;25(5):774-83.

14. Daley AJ, Bowden SJ, Rea DW, Billingham L, Carmicheal AR. What advice are oncologists and surgeons in the United Kingdom giving to breast cancer patients about physical activity? Int. J. Behav. Nutr. Phys. Act. 2008;5(1):46.

15. Pattinson L, Jessop A. The delivery of health improvement information during radiotherapy treatment: a survey of UK therapy radiographers. J Radiother Pract. 2016; 15 (2): 114–130

16. Williams K, Beeken RJ, Fisher A, Wardle J. Health professionals' provision of lifestyle advice in the oncology context in the United Kingdom. Eur J Cancer Care. 2015;24(4):522-30.

17. Koutoukidis DA, Lopes S, Fisher A, Williams K, Croker H, Beeken RJ. Lifestyle advice to cancer survivors: a qualitative study on the perspectives of health professionals. BMJ open. 2018;8(3).

18. Cantwell M, Walsh D, Furlong B, Moyna N, McCaffrey N, Boran L et al. Healthcare professionals' knowledge and practice of physical activity promotion in cancer care: Challenges and solutions. Eur J Cancer Care. 2018;27(2):12795.

19. Conlon K, Pattinson L, Hutton D. Attitudes of oncology healthcare practitioners towards smoking cessation: A systematic review of the facilitators, barriers and recommendations for delivery of advice and support to cancer patients. Radiography. 2017;23: 256-263

20. Sutton E, Hackshaw-McGeagh LE, Aning J, Bahl A, Koupparis A, Persad R et al. The provision of dietary and physical activity advice for men diagnosed with prostate cancer: a qualitative study of the experiences and views of health care professionals, patients and partners. Cancer Causes Control. 20171;28(4):319-29.

21. Spellman C, Craike M, Livingston PM. Knowledge, attitudes and practices of clinicians in promoting physical activity to prostate cancer survivors. Health Educ J. 2013:1– 10. 34.

22. Cancer Research UK. Vision for radiotherapy. 2014.

23. Halkett GK, Kristjanson LJ. Patients' perspectives on the role of radiation therapists. Patient Education and Counselling. 2007;69(1):76-83

For peer review only - http://bmjopen.bmj.com/site/about/guidelines.xhtml

24. Society and College of Radiographers (SCoR). The College of Radiographers Research Priorities for the Radiographic Profession. 2017

25. Chief Allied Heath Professions Officer's Team. Allied Health Professions into Action. Using Allied Health Professionals to transform health, care and wellbeing. 2017. NHS England

26. Allied Health Professions Federation. UK Allied Health Professions Public Health Strategic Framework 2019-2024. 2019. [Internet] Available at: http://www.ahpf.org.uk/files/UK%20AHP%20Public%20Health%20Strategic%20Framework%202019-2024.pdf. Accessed 17 Jan 2020.

27. NHS. An Implementation Guide and Toolkit for Making Every Contact Count: Using every opportunity to achieve health and wellbeing. 2016

28. Charlesworth L, Hutton D, Hussain H. Therapeutic Radiographers' perceptions of the barriers and enablers to effective smoking cessation support. Radiography. 2019;25(2):121-8.

29. Boaz, A, Baeza, J, Fraser, A. Effective implementation of research into practice: an overview of systematic reviews of the health literature. BMC research notes 2011; 4 (1): 212.

30. Gill P, Stewart K, Treasure E, and Chadwick B. Methods of data collection in qualitative research: interviews and focus groups. Br Dent J. 2008; 204(6):291-5.

31. Guest G, Bunce A, Johnson L. How many interviews are enough? An experiment with data saturation and variability. Field methods. 2006;18(1):59-82.

32. Saunders B, Sim J, Kingstone T, Baker S, Waterfield J, Bartlam B et al. Saturation in qualitative research: exploring its conceptualization and operationalization. Quality quantity. 2018;52(4):1893-907.

33. Gale NK, Heath G, Cameron E, Rashid S, Redwood S. Using the framework method for the analysis of qualitative data in multi-disciplinary health research. BMC medical research methodology. 2013;13(1):117.

34. Tong A, Sainsbury P, Craig J. Consolidated criteria for reporting qualitative research (COREQ): a 32-item checklist for interviews and focus groups. International journal for quality in health care. 2007;19(6):349-57.

35. Society of Radiographer. Radiotherapy radiographic workforce UK census 2018. [Internet] Available at https://www.sor.org/learning/document-library/radiotherapy-radiographic-workforce-uk-census-2018. Accessed 1 March 2020.

36. Williamson K, Williams KJ. Organisational justice, trust and perceptions of fairness in the implementation of agenda for change. Radiography. 2011;17(1):61-6.

For peer review only - http://bmjopen.bmj.com/site/about/guidelines.xhtml

37. McClinchy J, Williams J, Gordon L, Cairns M, Fairey G. Dietary Advice and Collaborative Working: Do Pharmacists and Allied Health Professionals Other Than Dietitians Have a Role? InHealthcare. 2015; 3(1):64-77. Multidisciplinary Digital Publishing Institute.

38. Scarborough P, Bhatnagar P, Wickramasinghe KK, Allender S, Foster C, Rayner M. The economic burden of ill health due to diet, physical inactivity, smoking, alcohol and obesity in the UK: an update to 2006–07 NHS costs. J. Public Health. 2011;33(4):527-35.

39. Thompson RL, Summerbell CD, Hooper L, Higgins JP, Little P, Talbot D et al. Dietary advice given by a dietitian versus other health professional or self-help resources to reduce blood cholesterol. Cochrane Database of Systematic Reviews. 2003(3).

40. The NHS. The NHS long term plan. 2019

41. Independent Cancer Taskforce. Achieving world-class cancer outcomes a strategy for England 2015-2020. 2015

42. Ruiz JG, Mintzer MJ, Leipzig RM. The impact of e-learning in medical education. Academic medicine. 2006; 81(3):207-12.

43. Childs S, Blenkinsopp E, Hall A, Walton G. Effective e-learning for health professionals and students—barriers and their solutions. A systematic review of the literature—findings from the HeXL project. Health Information & Libraries Journal. 2005;22:20-32.

44. Cook DA, Levinson AJ, Garside S, Dupras DM, Erwin PJ, Montori VM. Internet-based learning in the health professions: a meta-analysis. Jama. 2008;300(10):1181-96.

45. Gray-Burrows KA, Willis TA, Foy R, Rathfelder M, Bland P, Chin A, Hodgson S, Ibegbuna G, Prestwich G, Samuel K, Wood L. Role of patient and public involvement in implementation research: a consensus study. BMJ quality & safety. 2018;27(10):858-64.

46. Avery KN, Donovan JL, Horwood J, Neal DE, Hamdy FC, Parker C et al. The importance of dietary change for men diagnosed with and at risk of prostate cancer: a multi-centre interview study with men, their partners and health professionals. BMC family practice. 2014;15(1):81.

47. Marmot M, Atinmo T, Byers T, Chen J, Hirohata T, Jackson A et al. Food, nutrition, physical activity, and the prevention of cancer: a global perspective. Office for national statistics. 2019. Cancer registration statistics, England: 2017.

48. National Cancer Registration & Analysis Service and Cancer Research UK: Chemotherapy, Radiotherapy and Tumour Resections in England: 2013-2014 workbook. London: NCRAS; 2017.

For peer review only - http://bmjopen.bmj.com/site/about/guidelines.xhtml

Supplementary material 1: Interview topic guide

Topic	Question	Prompt
**Introduction**	Brief introductions; get verbal consent to tape-record the interview; remind the aims of study, check length of interview/audio-recorded.	
**Health promotion beliefs**	What are your views on the role of health behaviours in those with a diagnosis of cancer?<br><br>Do you think delivering advice on improving health behaviours is part of your role?	Breast/prostate/colorectal/head & neck cancers<br><br>Healthy eating/ exercise/ alcohol/ smoking / weight management
**Provision of advice on healthy behaviours**	Can you tell me about your experiences of providing health behaviour advice to cancer patients?<br><br>Are there some health behaviours you recommend more than others?<br><br>Why do you think that is?<br><br>Are there some patient groups that you provide health behaviour advice to more than others?<br><br>Why do you think that is?	Healthy eating/ exercise/ alcohol/ smoking / weight management
**Barriers to health advice delivery**	On the survey we asked you about a number of barriers limiting you in providing health behaviour advice to your patients. You mentioned X Y Z<br><br>Can you tell me more about these barriers?<br><br>Can you think of any other barriers within your role and work environment stopping you from giving lifestyle and health promotion advice to patients?	Why do you think that is?
**Addressing barriers**	What support do you think would be most helpful in enabling therapeutic radiographers to deliver health behaviour advice to patients?	
**Training preferences**	How helpful do you think an online training course specifically for therapeutic radiographers on delivering health behaviour advice to cancer patients would be?<br><br>How helpful do you think a face to face training course specifically for therapeutic radiographers on delivering health behaviour advice to cancer patients would be?<br><br>If you were provided with a training course (online or face to face) what topics would you like delivered?	Why do you think this is?
**Finish**	Thank you for your time. Do you have anything else you would like to add?	

For peer review only

COREQ (COnsolidated criteria for REporting Qualitative research) Checklist

A checklist of items that should be included in reports of qualitative research. You must report the page number in your manuscript where you consider each of the items listed in this checklist. If you have not included this information, either revise your manuscript accordingly before submitting or note N/A.

Topic	Item No.	Guide Questions/Description	Reported on Page No.
**Domain 1: Research team and reflexivity**			
Personal characteristics			
Interviewer/facilitator	1	Which author/s conducted the interview or focus group?	
Credentials	2	What were the researcher's credentials? E.g. PhD, MD	
Occupation	3	What was their occupation at the time of the study?	
Gender	4	Was the researcher male or female?	
Experience and training	5	What experience or training did the researcher have?	
Relationship with participants			
Relationship established	6	Was a relationship established prior to study commencement?	
Participant knowledge of the interviewer	7	What did the participants know about the researcher? e.g. personal goals, reasons for doing the research	
Interviewer characteristics	8	What characteristics were reported about the inter viewer/facilitator? e.g. Bias, assumptions, reasons and interests in the research topic	
**Domain 2: Study design**			
Theoretical framework			
Methodological orientation and Theory	9	What methodological orientation was stated to underpin the study? e.g. grounded theory, discourse analysis, ethnography, phenomenology, content analysis	
Participant selection			
Sampling	10	How were participants selected? e.g. purposive, convenience, consecutive, snowball	
Method of approach	11	How were participants approached? e.g. face-to-face, telephone, mail, email	
Sample size	12	How many participants were in the study?	
Non-participation	13	How many people refused to participate or dropped out? Reasons?	
Setting			
Setting of data collection	14	Where was the data collected? e.g. home, clinic, workplace	
Presence of non-participants	15	Was anyone else present besides the participants and researchers?	
Description of sample	16	What are the important characteristics of the sample? e.g. demographic data, date	
Data collection			
Interview guide	17	Were questions, prompts, guides provided by the authors? Was it pilot tested?	
Repeat interviews	18	Were repeat inter views carried out? If yes, how many?	
Audio/visual recording	19	Did the research use audio or visual recording to collect the data?	
Field notes	20	Were field notes made during and/or after the inter view or focus group?	
Duration	21	What was the duration of the inter views or focus group?	
Data saturation	22	Was data saturation discussed?	
Transcripts returned	23	Were transcripts returned to participants for comment and/or	

For peer review only

Topic	Item No.	Guide Questions/Description	Reported on Page No.
		correction?	
**Domain 3: analysis and findings**			
Data analysis			
Number of data coders	24	How many data coders coded the data?	
Description of the coding tree	25	Did authors provide a description of the coding tree?	
Derivation of themes	26	Were themes identified in advance or derived from the data?	
Software	27	What software, if applicable, was used to manage the data?	
Participant checking	28	Did participants provide feedback on the findings?	
Reporting			
Quotations presented	29	Were participant quotations presented to illustrate the themes/findings? Was each quotation identified? e.g. participant number	
Data and findings consistent	30	Was there consistency between the data presented and the findings?	
Clarity of major themes	31	Were major themes clearly presented in the findings?	
Clarity of minor themes	32	Is there a description of diverse cases or discussion of minor themes?	

Developed from: Tong A, Sainsbury P, Craig J. Consolidated criteria for reporting qualitative research (COREQ): a 32-item checklist for interviews and focus groups. *International Journal for Quality in Health Care*. 2007. Volume 19, Number 6: pp. 349 – 357

Once you have completed this checklist, please save a copy and upload it as part of your submission. DO NOT include this checklist as part of the main manuscript document. It must be uploaded as a separate file.

For peer review only

BMJ Open

Therapeutic radiographers' delivery of health behaviour change advice to those living with and beyond cancer: a qualitative study.

Journal:	*BMJ Open*
Manuscript ID	bmjopen-2020-039909.R1
Article Type:	Original research
Date Submitted by the Author:	04-Jun-2020

Complete List of Authors:	Pallin, Nickola; University College London, Behavioural Science and Health; London South Bank University, Department of Allied Health Sciences<br>Beeken, Rebecca; University College London, Behavioural Science and Health; University of Leeds Leeds Institute of Health Sciences<br>Pritchard-Jones, Kathy; London Cancer<br>Charlesworth, Laura; University of Lincoln<br>Woznitza, Nicholas; Homerton University Hospital NHS Foundation Trust, Radiology; Canterbury Christ Church University, Canterbury Christ Church University<br>Fisher, Abigail; University College London, Behavioural Science and Health

**Primary Subject Heading**:	Oncology
Secondary Subject Heading:	Public health
Keywords:	RADIOTHERAPY, MEDICAL EDUCATION & TRAINING, Adult oncology < ONCOLOGY, QUALITATIVE RESEARCH

SCHOLARONE™ Manuscripts

I, the Submitting Author has the right to grant and does grant on behalf of all authors of the Work (as defined in the below author licence), an exclusive licence and/or a non-exclusive licence for contributions from authors who are: i) UK Crown employees; ii) where BMJ has agreed a CC-BY licence shall apply, and/or iii) in accordance with the terms applicable for US Federal Government officers or employees acting as part of their official duties; on a worldwide, perpetual, irrevocable, royalty-free basis to BMJ Publishing Group Ltd ("BMJ") its licensees and where the relevant Journal is co-owned by BMJ to the co-owners of the Journal, to publish the Work in this journal and any other BMJ products and to exploit all rights, as set out in our licence.

The Submitting Author accepts and understands that any supply made under these terms is made by BMJ to the Submitting Author unless you are acting as an employee on behalf of your employer or a postgraduate student of an affiliated institution which is paying any applicable article publishing charge ("APC") for Open Access articles. Where the Submitting Author wishes to make the Work available on an Open Access basis (and intends to pay the relevant APC), the terms of reuse of such Open Access shall be governed by a Creative Commons licence – details of these licences and which Creative Commons licence will apply to this Work are set out in our licence referred to above.

Other than as permitted in any relevant BMJ Author's Self Archiving Policies, I confirm this Work has not been accepted for publication elsewhere, is not being considered for publication elsewhere and does not duplicate material already published. I confirm all authors consent to publication of this Work and authorise the granting of this licence.

For peer review only

Title: Therapeutic radiographers' delivery of health behaviour change advice to those living with and beyond cancer: a qualitative study.

Authors

Pallin N D [1, 2] Beeken R J [1,3] Pritchard Jones K [4] Charlesworth L[5] Woznitza N[6] Fisher A[1]

1. Department of Behavioural Science and Health, University College London, London, UK
2. Department of Allied Health Sciences, London South Bank University, London, UK.
3. Leeds Institute of Health Sciences, University of Leeds, Leeds, UK
4. London Cancer, London, UK
5. College of Social Science, University of Lincoln, Lincolnshire, UK.
6. Radiology Department, Homerton University Hospital, London, UK.

Nickola Pallin email: pallinn@lsbu.ac.uk

Dr Rebecca Beeken email: R.Beeken@leeds.ac.uk

Prof Kathy Pritchard Jones: k.pritchard-jones@ucl.ac.uk

Laura Charlesworth: LCharlesworth@lincoln.ac.uk

Dr Nick Woznitza: Nicholas.woznitza@nhs.net

Dr Abi Fisher email: Abigail.fisher@ucl.ac.uk

Present address of corresponding author: Nickola Pallin

School of Health and Social Care

Department of Allied Health Sciences

London South Bank University

103 Borough Road

London

SE1 0AA

Tel: 0207815674

Email: pallinn@lsbu.ac.uk

Note: This work was undertaken in the Department of Behavioural Science and Health, University College London, London, UK. The lead author was based there as a pre-doctoral research fellow.

For peer review only - http://bmjopen.bmj.com/site/about/guidelines.xhtml

Abstract

Objectives Therapeutic radiographers (TRs) are well placed to deliver health behaviour change advice to those living with and beyond cancer (LWBC). However, there is limited research on the opinions of TRs around delivering such advice to those LWBC.

Setting and Participants This qualitative study aimed to explore therapeutic radiographers' practices and facilitators in delivering advice on physical activity, healthy eating, alcohol intake, smoking and weight management. Fifteen UK-based therapeutic radiographers took part in a telephone interview using a semi-structured interview guide.

Data was analysed using the framework analysis method.

Results Emergent themes highlighted that TRs are mainly aware of the benefits of healthy behaviours in managing radiotherapy treatment related side effects, with advice provision lowest for healthy eating and physical activity. Participants identified themselves as well placed to deliver advice on improving behaviours to those LWBC, however reported a lack of knowledge as a limiting factor to doing so. The TRs reported training and knowledge as key facilitators to the delivery of advice, with a preference for online training.

Conclusions There is a need for education resources, clear referral pathways and in particular training for TRs on delivering physical activity and healthy eating advice to those LWBC.

For peer review only - http://bmjopen.bmj.com/site/about/guidelines.xhtml

Strengths and limitations of this study

- This study provides insight in therapeutic radiographers' views on all key modifiable health behaviours for those living with and beyond cancer.
- The participants worked in different radiotherapy departments, offering insight into the practices among therapeutic radiographers in the delivery of healthy behaviour advice from a wide range of hospitals.
- Whilst the sample size was small data saturation was reached.
- Participants might be more interested in the role of health behaviours in cancer survivorship, which might bias the responses towards a positive view on the role of therapeutic radiographers' in delivering advice within their role.

Keywords: Therapeutic Radiographer, cancer, health behaviours, healthy eating, physical activity, lifestyle, behaviour change

For peer review only

Introduction

It is estimated that 40% of cancer cases are linked to unhealthy behaviours [1]. Based on evidence from systematic literature reviews and meta analyses, the World Cancer Research Fund (WCRF) recommend that individuals are physically active, limit consumption of energy dense foods, salty foods, red meat and avoid processed meat, eat more plant foods, maintain a healthy weight, limit alcoholic drinks and avoid tobacco to reduce their risk of cancer [2]. Those living with and beyond cancer (LWBC) are also advised to follow these guidelines, due to increasing evidence that healthy behaviours may improve physical and psychosocial outcomes after a cancer diagnosis [2 - 8].

Despite the potential benefits of healthy behaviours, few people LWBC are meeting the WCRF recommended health behaviour recommendations [9, 10]. Those LWBC report one key reason for not adopting healthier lifestyle behaviours is lack of advice and support from their healthcare team [11]. Healthcare professionals (HCPs) are well placed to bring about positive health behaviour changes among cancer patients [12]. A trial of brief advice among breast cancer survivors showed that a simple physical activity recommendation from a HCP doubled the percentage meeting national exercise guidelines [12]. Despite this, research to date among both HCPs and those LWBC consistently shows that few oncology HCPs offer guidance to oncology patients on healthy lifestyle behaviours [13 – 21].

Reported barriers among HCPs in providing health behaviour advice for those LWBC, include believing that giving advice was not part of their role, lack of time with patients, lack of referral programmes, lack of resources such as education leaflets for those LWBC, and lack of knowledge regarding guidelines and research findings [16 – 21]. A recent qualitative study with 21 oncology HCPs identified that advice on health behaviours provided to those LWBC focused on general health and controlling side effects, with few HCPs advising on health behaviours in the context of improving survival outcomes [20]. Whilst these studies provide useful insight into the practices and barriers among oncology HCPs the participants within these studies were primarily oncologists and nurses and

For peer review only - http://bmjopen.bmj.com/site/about/guidelines.xhtml

focused on the provision of physical activity and weight management advice. There is limited research on the opinions of therapeutic radiographers (TRs) in delivering advice on health behaviours to those LWBC. Despite at least 50% of cancer patients receiving radiotherapy as part of their cancer treatment [22].

TRs are the only health professionals qualified to deliver radiotherapy and play a central role in supporting cancer patients [23]. In the UK, the College of Radiographers recognise the importance of TRs in providing health behaviour advice to improve patient outcomes [24]. TRs are also seen as an integral part of the health force in driving improvements in wellbeing as outlined in the 2017 publication of "AHPs into Action, using Allied Health Professionals to transform health, care and wellbeing" which states that radiographers are key to implementing a preventative healthcare approach and that their expertise should be used to design and deliver health interventions [25]. TRs are ideally placed to deliver health behaviour advice, particularly through Making Every Contact Count (MECC) [26,27.] MECC is a strategy whereby health professionals use every appropriate opportunity and interaction with patients to promote healthy behaviours and signpost to relevant healthcare services using an 'Ask, Advise, Act' framework [27]. MECC fits extremely well within the TRs' role in which patient education is a key part of radiotherapy practice with TRs providing care to the same patient every day, often over a number of weeks [23]. TRs therefore have the potential to make significant contributions in supporting positive health behaviour changes among those LWBC.

However, despite these opportunities one survey in the UK among 102 TRs identified that TRs rarely advise patients on the key modifiable health behaviours including smoking, alcohol, healthy eating and exercise [15]. The findings also showed lack of knowledge and training as barriers among TRs in delivering advice on these topics [15]. Similarly, focus group interviews with 38 TRs identified that lack of knowledge and training were barriers to the provision of smoking cessation advice [28].

For peer review only - http://bmjopen.bmj.com/site/about/guidelines.xhtml

Challenges remain in translating behaviour change interventions into existing care pathways and practices in a way that is appropriate for use by health professionals [29]. Understanding TRs' practices, and what support they need in delivering advice on the topics of physical activity, healthy eating, alcohol intake, smoking and weight management could inform the development of interventions that will enable TRs' in delivering advice on improving health behaviours to those LWBC. Qualitative research is appropriate for exploring the beliefs, experiences and motivations of individuals on specific matters and allow for more information and clarification [30]. Limited qualitative data exists on TRs' practices and views on delivering advice on these health behaviour topics. This study therefore aimed to address this and through a qualitative methodology explore TRs' practices in delivering health behaviour advice, in addition to exploring the facilitators in delivering such advice. Preferences regarding training on delivering this advice were also explored.

Methods

Participants and Recruitment

Participants were TRs working in the UK in a clinical role. They had provided their contact details on a previous online survey investigating TR's practices in delivering health behaviour advice agreeing to be invited for a follow up telephone interview. An email was sent with an information sheet explaining the research and inviting these TRs. Those who agreed to take part signed a consent form prior to the telephone interview. Ethical approval was granted by the University College London Research Ethics Committee, reference 12945/001.

Data collection

Semi-structured individual telephone interviews were carried out between April and May 2019 by a lecturer in therapeutic radiography with an MSc who had completed qualitative interviewing as part of their training (NP). The interviewer had no previous relationship with the study participants. The

For peer review only - http://bmjopen.bmj.com/site/about/guidelines.xhtml

topic guide (supplementary material 1) was based on the guide used within a previous study [17] which explored oncology HCPs views on the provision of lifestyle advice to cancer patients. This guide was adapted for use among TRs, with additional questions added to assess preferences for training on delivering advice. The topic guide was piloted with two participants to check for comprehension of the questions. This data was included in the analysis because no substantial changes were required. The interviews lasted approximately 30 min (range: 20 – 40 min) and were audio-recorded, anonymised and transcribed verbatim. The transcripts were verified by NP against each recording to confirm accuracy. The aim was to carry out interviews until data saturation was reached. It was anticipated that ten participants would be required to reach data saturation because it was a homogeneous group [31]. After ten interviews were carried out, they were transcribed verbatim. Following familiarisation with the data NP generated the initial codes and it appeared that saturation was reached after ten interviews as no new codes occurred in the tenth interview [32]. A further five interviews were carried out to confirm this.

Patient and public involvement

Patient input was not used in the design of the research methods. However, the topic guide was piloted with TRs in the academic setting. Additionally, the topic guide was piloted with two participants and these were included in the analysis.

Analysis

The interview transcripts were analysed using the framework analysis method [33]. This method was chosen because it is an appropriate method for analysing homogenous data and semi-structured interview transcripts, it is also appropriate when using inductive qualitative analysis [33]. A random selection of transcripts (n = 3) were independently coded by AF to check for reliability. The researchers NP, AF and RJB met and agreed on a final coding list in an iterative process (AF and RJB are both

For peer review only - http://bmjopen.bmj.com/site/about/guidelines.xhtml

experienced qualitative researchers and health psychologists). These agreed set of codes formed the analytical framework which was then applied to all of the transcripts and the data summarised in a matrix using Microsoft excel. Themes were generated by reviewing the matrix connecting the data between the participants and the codes. The completed consolidated criteria for reporting qualitative research checklist is available in the supplementary material 2 [34]. Themes are presented in the results with supporting quotes and the participants identifier (Table 1).

Table 1: Participant identifier and demographic characteristics

Participant Identifier	Demographic characteristics	
	**Gender**	**Professional Grade**
TR 1	Female	Band 6
TR 2	Female	Band 7
TR 3	Female	Band 7
TR 4	Female	Band 7
TR 5	Female	Band 7
TR 6	Female	Band 7
TR 7	Female	Band 7
TR 8	Male	Band 8
TR 9	Female	Band 7
TR 10	Female	Band 7
TR 11	Female	Band 6
TR 12	Female	Band 7
TR 13	Male	Band 5
TR 14	Female	Band 7
TR 15	Male	Band 8

TR: Therapeutic Radiographer

Results

Participants

The radiotherapy radiography workforce census in the UK only reports the workforce's professional grade, and no other demographics [35]. In the UK, TRs' level of professional skills and knowledge are categorised by agenda for change (AfC) professional band grades 5 to 8 [36]. Therefore, in this study,

For peer review only - http://bmjopen.bmj.com/site/about/guidelines.xhtml

the participants gender and professional grade were collected; no other demographic information was collected (Table 1). Seventy-two TRs were emailed and invited to take part in an interview, 15 returned consent forms and completed the telephone interview. Fifteen interviews were conducted with 12 women and 3 men. The participants came from all regions of the UK, including England, Wales, Scotland and Northern Ireland.

Themes

Five main themes were identified: (1) TRs provide behaviour change advice to manage radiotherapy related side effects (2) TRs make judgements about when it is appropriate to deliver health behaviour advice (3) Knowledge and training are key facilitators in the delivery of health behaviour advice (4) TRs feel patients undergoing radiotherapy treatment seek guidance on health behaviours (5) TRs identify themselves as well placed to give health behaviour advice to patients.

TRs provide behaviour change advice to manage radiotherapy related side effects

Most respondents reported that they only provided advice on health behaviours that they believed would minimise radiotherapy related side effects. This meant smoking cessation and alcohol intake were the two health behaviours TRs mainly advised on.

"With head and neck patients, we give advice, particularly on smoking and drinking, obviously get worse side effects" (TR 6).

"The only thing we do generally say is about drinking plenty of fluids, avoiding alcohol. But that's more to do with prostate side effects, bladder reactions, and reducing gas" (TR 14).

"Radiographer are comfortable talking about alcohol when it comes to managing side effects" (TR 12).

For peer review only - http://bmjopen.bmj.com/site/about/guidelines.xhtml

No TRs reported advising patients on healthy eating. Some TRs mentioned advising patients on dietary intake but this is to patients who are at risk of losing weight, for side effect management and potential impact on accuracy of radiotherapy treatment delivery.

"Healthy eating, I don't tend to discuss too much. A lot of patients have difficulty eating and we are encouraging maintaining weight while on treatment" (TR 5).

"I'm not very sure if healthy eating is important. Any patients where we're treating, lower GI or pelvis, we would advise them to avoid very high-fibre foods, spicy foods, that might make them have very loose bowels, but other than that we say more or less keep on your same diet. We wouldn't generally discuss a healthy diet as a standard for all patients. No" (TR 15).

Some TRs mentioned they advise patients to be physically active, however this was only in the context of managing radiotherapy and cancer related fatigue.

"So exercise is one of my main ones that I focus on with all patients, particularly to help with their fatigue" (TR 9).

"Exercise, I say that's its quite beneficial to help with fatigue" (TR 12).

"I guess when we have patients come in, fatigue is one of the side effects, so we encourage our patients to remain active" (TR 15).

TRs make judgements about when it is appropriate to deliver health behaviour advice

TRs explained only discussing health behaviours, particularly smoking and alcohol with patients if there were evident indications of a problem. TRs also often reported making a judgment of whether

For peer review only - http://bmjopen.bmj.com/site/about/guidelines.xhtml

appropriate to advise a patient on a particular health behaviour.

"So, quite often you can tell if a patient is a smoker, you can smell it, or you can tell by their skin" (TR 11).

"I tend to give advice when you make a judgement of when it's appropriate, an example might be if a patient smell of smoke" (TR 12).

"Had patients come in and will smell of alcohol and at that time I'll say to the patient that it can exacerbate side effects" (TR 15).

This meant TRs did not provide advice on health behaviours to every patient.

"But for those patients where it's not clearly going to benefit them to stop drinking, you would just mention it very briefly. Not every patient will have that information" (TR 5).

Knowledge and training are keys facilitator in the delivery of health behaviour advice

Delivery of advice matched by knowledge

The reported delivery of advice on health behaviours appeared to be matched by knowledge of the benefits among those LWBC.

One participant explained how he only appreciated the importance of PA in cancer survivorship after attending a talk and being made aware of the evidence.

"My experience of appreciating the role exercise was from attending a talk. I suppose it was really just

For peer review only - http://bmjopen.bmj.com/site/about/guidelines.xhtml

highlighting in the studies the benefits obviously of a healthy lifestyle and introducing physical activity for patients on treatment" (TR 15).

Healthy eating was a topic the participants felt particularly unqualified to deliver advice on, and reported lack of knowledge as a barrier to the delivery of advice on healthy eating.

"It's a difficult one, diet, I think. It's more a knowledge thing. If you don't have the knowledge about what you can and can't say, you're just not going to approach the subject" (TR 12).

A need for continuous post graduate online training

All interviewed said they would welcome post graduate training on delivering health behaviour advice. The majority expressed a preference for online training to help overcome the barrier of limited time among TRs to attend training.

"Online, you're not having to take time out of clinical practice, online is more accessible" (TR 6)

Participants also mentioned that online training allows for yearly updates and continuous professional development (CPD).

"I think it'd be good (online training) because you can do it in your own time. Because I think that's sometimes the problem. You have this training once and then maybe it never gets brought up again. So it would be quite handy to have something small, every year, alongside all your other mandatory training" (TR 12).

Participants did acknowledge that face to face training allows for further questioning that's not possible with online training.

For peer review only - http://bmjopen.bmj.com/site/about/guidelines.xhtml

"I think one to one training, because you can ask questions that may not be covered within the online training" (TR 1).

To overcome of the barrier of not all staff being able attend face to face training participants suggested it would be useful to train some TRs through face to face methods that they could then cascade to other TRs within the radiotherapy department.

"Maybe some face-to-face with some staff, that they could cascade down, might be useful as well" (TR 4).

A need for training in the undergraduate setting

It was also suggested to incorporate training on delivering lifestyle advice into the undergraduate education program.

"Certainly, get it into the undergraduate course to start with, making them aware it is part of the role" (TR 9).

"It's still not something that I can say was primarily covered in the undergraduates' training about the benefits of healthy lifestyle, you know, there's no real formal education that I can see" (TR 15).

TRs reported knowledge of resources and referral pathways as facilitators in the delivery of advice

Participants also felt knowledge of how to refer patients onto further support would enable them to have conversations on improving health behaviours. With some TRs reporting that lack of knowledge of resources and referral pathways are barriers to initiating a conversation on behaviour change.

For peer review only - http://bmjopen.bmj.com/site/about/guidelines.xhtml

For peer review only

"There needs to be more information available to professionals of where exactly you can refer patients to, whether that be website, whether that be app" (TR 13).

"That's the only reason why they (therapeutic radiographers) don't want to open these conversations up, because they don't know where to go with it or how to refer on" (TR 9).

They also acknowledge that in the short time they have with patients if they had a resource then would be more inclined to advise.

"Having something on a piece of paper, education and having the resources, if you can do it in two minutes you should be able slip that in" (TR 2).

"You don't always have that information at hand, so if it was readily available, I think we'd give out a bit more (health behaviour information) if it was just the case of pointing them in the right direction that would be a quick and easy thing to do" (TR 3).

The benefit of incorporating patients' perspective into training

Participants also mentioned that getting patients' perspectives on receiving advice on improving health behaviours should be incorporated into training.

"I think that would be better coming from the patients themselves, rather than just feedback from what journals, and other literature says" (TR 7).

"If there'd even be patients that would be willing to maybe just even be involved with staff training" (TR 3).

For peer review only - http://bmjopen.bmj.com/site/about/guidelines.xhtml

TRs feel patients undergoing radiotherapy treatment seek guidance on health behaviours.

Many of the TRs also described that patients often ask them for guidance around health behaviour changes, particularly on diet and exercise. This shows that patients see TRs as credible sources of information on health behaviours.

"We are getting asked the question more and more about weight loss, healthy living, wanting to exercise more" (TR 4).

"It is quite a common thing to be asked at the end of treatment, not so much the smoking and alcohol, I have to say, but diet and exercise is certainly something that people commonly ask" (TR 3).

TRs identify themselves as well placed to give health behaviour advice to patients.

TRs acknowledged that they are a consistent healthcare member for patients undergoing radiotherapy and have many opportunities to deliver lifestyle advice. Therefore, TRs recognised that they are well placed to deliver health behaviour advice to patients.

"We're in a unique position because we do see the same patient day after day and you do kind of start to develop a relationship with them" (TR 10).

"I think we're well placed to help influence patients' behaviours and it' something we should be seen to encourage and report" (TR 7).

"We're in the best position where we see the patients, for a number of weeks every day to encourage any changes" (TR 8).

From the interviews it appeared that many patients undergoing radiotherapy, excluding those at risk

For peer review only - http://bmjopen.bmj.com/site/about/guidelines.xhtml

of malnutrition or significant weight loss, are primarily reviewed and assessed by TRs. This highlights that TRs are in an ideal position to deliver advice on health behaviours, particularly when asked about nutrition advice delivery.

"They routinely see the specialist radiographer, for the breast patients. But they don't have a dietitian appointment" (TR 12).

"Prostate and breast are two tumour groups that are fully radiographer-led review and about 70% to 80% of our work load. They generally wouldn't be sent to a dietician" (TR 15).

"Only have a dietitian on board for the head and necks" (TR 9).

Discussion

TRs in this study saw themselves as well placed to deliver health behaviour advice, but also reported that they do not routinely provide advice to all patients. TRs were particularly unlikely to provide advice on healthy eating and physical activity, and were more likely to provide advice on those behaviours they believed would minimise radiotherapy or cancer related side effects. This is in line with previous research among TRs [15,28,37]. In one qualitative study a key facilitator reported among TRs in delivering smoking cessation support to patients was knowledge of the link between smoking and toxicity [28]. Another qualitative study that explored allied health professionals' views regarding the provision of dietary advice to patients, highlighted that TRs report giving dietary advice to help counteract the side effects of radiotherapy [37]. Additionally, in our study, if TRs did provide dietary advice, this tended to be general advice rather than cancer-specific advice on healthy eating.

In some studies, oncology HCPs have reported they do not self-identify as the right person to provide lifestyle advice [17,20]. However, in this study TRs identified themselves as being well placed to deliver

For peer review only - http://bmjopen.bmj.com/site/about/guidelines.xhtml

health behaviour advice and in a unique position as a consistent member of the multidisciplinary team providing care to patients. However, despite this, they do not feel qualified to deliver advice, particularly on the topic of healthy eating. In the UK, poor diet has the biggest impact on the NHS budget, greater than alcohol consumption, smoking and physical inactivity [38]. It has been noted that there are insufficient dietitians to provide dietary advice to all patients who may need dietary support [39]. In response to this all HCPs are being asked to implement a preventative healthcare approach within their role and the delivery of healthy eating advice is fundamental to this [23,24,40,41]. Key to achieving this is that TRs will have the skills, knowledge and behaviours to improve the health and wellbeing of individuals [24]. As with other oncology HCP groups [16,17,20,37] this study identifies the need for education and training among TRs in delivering health behaviour advice, particularly on healthy eating and physical activity. This training should also address when and how to refer to other support if necessary, as this was identified as a key facilitator in the delivery of advice on health behaviours, particularly when time is a barrier to the delivery of this advice [15,16,17].

All interviews demonstrated that TRs would welcome training on delivering health behaviour advice and recommended it as a key facilitator in delivering advice, in addition to incorporating it into the undergraduate setting. The need for postgraduate training among TRs in delivering health advice has also recently been reported by Charlesworth et al. [28] in relation to the delivery of smoking cessation advice. Our findings from this study provide additional insight into TRs preferences on the type of training on delivering lifestyle advice to those LWBC, with TRs demonstrating a preference of online training in the postgraduate setting. Amongst HCPs online education has been reported to be as effective as face-to-face education [42]. Additionally, the use of online learning enables HCPs to carry out training at time that fits in with clinical work [43, 44]. TRs in this study identified this benefit of online learning in overcoming the limited time available for TRs to undertake continuous professional development and additional training. Interestingly, TRs in this study mentioned having patient input in the training would be helpful. Whilst HCPs input is key to the development of interventions, patient

For peer review only - http://bmjopen.bmj.com/site/about/guidelines.xhtml

members play key advocacy roles and their input can enhance the outcomes of interventions [45]. Patient input may also help overcome the reported barrier of fear of causing offence to a patient, which has been reported as a barrier amongst oncology HCPs in delivery of health behaviour advice [17].

Those LWBC wish to receive advice on health behaviours from their healthcare team [13,20,46] and is of particular importance as the period following a cancer diagnosis has been shown be a teachable moment and an ideal opportunity to motivate patients around the importance of healthy eating and physical activity [47,48]. This was made apparent in this study, whereby some TRs mentioned that healthy eating and exercise were the health behaviours patients ask for advice on more often, generally towards the end of their treatment. This further highlights the importance of supporting TRs in delivering evidence-based health behaviour advice to meet patients' needs.

TRs have a responsibility to educate patients on the importance of following healthy behaviours given the increasing evidence showing implementing healthy behaviours improve a number of physical and psychosocial outcomes after a cancer diagnosis [2,3]. Among pre- and post-menopausal women living with and beyond breast cancer, a systematic literature review and meta-analysis of 82 follow-up studies (n = 213075 breast cancer survivors) identified that being overweight increases the risk of all cause and breast cancer mortality [4]. Being physically active after a cancer diagnosis is also correlated with improved survival and reduced recurrence [5,6,49]. Whilst data is limited, emerging research suggests healthy dietary behaviours after a diagnosis may improve outcomes [3,50]. In a prospective observational study of 1009 patients with stage III colon cancer, a higher intake of a typical Western diet was associated with a three-fold increased risk of disease recurrence and a 2.3-fold increased risk of all-cause mortality [8]. Additionally, those LWBC are at increased risk for developing cardiovascular disease, osteoporosis, and diabetes and healthy behaviours can reduce the risk of developing these diseases [51,52].

For peer review only - http://bmjopen.bmj.com/site/about/guidelines.xhtml

Of those interviewed in this study it appeared that those with breast, prostate and colorectal cancer are primarily reviewed and assessed by TRs. Therefore, it is the responsibility of TRs to deliver advice on improving health behaviours to these patients. This is also particularly important because the strongest evidence for the benefits of diet and exercise is currently in breast, prostate and colorectal cancer survivors [53]. These are also the most common cancers in the UK and radiotherapy plays a key role in managing these cancers [22, 54]. Therefore, with the right skills and knowledge, TRs could deliver advice on improving health behaviours. By supporting self-efficacy among patients towards the end of their treatment which very often is in the radiotherapy department can be empowering for patients. Among those with prostate cancer, implementing dietary changes brought psychological benefit, as a method of coping and regaining control over their diagnosis [46].

Strengths and limitations

This is the first qualitative study among TRs to explore the provision of advice on all key modifiable lifestyle behaviours for those LWBC as per recommendations [2]. Whilst the aim of qualitative research is not to generalise the findings the sample size was small, and therefore the findings may not be representative of the views of the wider therapeutic radiography workforce. However, data saturation was reached, likely due the homogeneous sample of participants. Additionally, the participants worked in different radiotherapy departments and therefore provide insight into the practices among TRs in the delivery of healthy behaviour advice from a wide range of hospitals. Also, the participants worked in cancer centres in England, Wales, Scotland and Northern Ireland, providing insight into the practices across the UK. A limitation may be that the participants might be more interested in the role of health behaviours in cancer survivorship, which might bias the responses towards a positive view on this topic and the role of TRs in delivering advice within their role. Despite this however, provision of health behaviour change advice was low, suggesting TRs may be even less likely to educate patients around the importance of healthy behaviours.

For peer review only - http://bmjopen.bmj.com/site/about/guidelines.xhtml

Future research

This study highlights the need for training and education among TRs on the delivery of health behaviour advice to cancer patients, both in the undergraduate and postgraduate setting. Particularly on the topics of physical activity, healthy eating and weight management. Higher Education Institutions have a responsibility in educating the Allied Health Professional workforce on implementing health promotion within their role [55]. Further research among pre-registration TR students and lecturers within therapeutic radiography should therefore explore how best to address this need. Future research among TRs should also use purposive sampling to identify the views and health promotion practices among those who may not have a primary interest in the area of health behaviours among those LWBC.

Conclusion

In conclusion, whilst the majority of TRs delivered some advice on health behaviours as part of their role, advice was mainly on smoking and alcohol intake. Most believed in the value of this advice in managing radiotherapy and cancer related side effects. Provision of advice was lowest for weight management, healthy eating and physical activity. The findings show a need for training among TRs in delivering advice on improving health behaviours among those LWBC, with TRs reporting a preference for online training in the post graduate setting.

Declarations

Contributors: NDP had the original idea for the study and obtained the funding with AF, RJB and KPJ. NDP developed the design of the study, acquired the data, analysed and interpreted the data, drafted and revised the article and approved the final the manuscript submitted. AF provided behavioural science expertise, contributed to the development of the study design, the recruitment approach, analysed and interpreted the data, reviewed, edited and approved the final manuscript. RJB provided

For peer review only - http://bmjopen.bmj.com/site/about/guidelines.xhtml

behavioural science expertise, contributed to the development of the study design, the recruitment approach, interpreted the data and reviewed, edited and approved the final manuscript. KPJ provided oncology expertise and intellectual input into the recruitment approach, design and approved the final manuscript. LC provided therapeutic radiography and public health expertise which informed the development of the study design and reviewed the article and approved the final manuscript. NW provided radiography and public health expertise and contributed to the development of the study design, reviewed the manuscript for important intellectual content and approved the final the manuscript submitted.

Competing Interests: None

Funding: This research was funded by Cancer Research UK (ref no: C59769/A25965).

Data sharing statement: Data can be obtained by the corresponding author on reasonable request.

Ethics approval: The methodology for this study was approved by the Human Research Ethics committee of University College London (reference 12945/001).

Consent to participate: Informed consent was obtained from all individual participants included in the study.

Acknowledgments: The researchers are grateful to the health professionals who participated in the study.

For peer review only - http://bmjopen.bmj.com/site/about/guidelines.xhtml

References

1. Brown KF, Rumgay H, Dunlop C, Ryan M, Quartly F, Cox A et al. The fraction of cancer attributable to modifiable risk factors in England, Wales, Scotland, Northern Ireland, and the United Kingdom in 2015. Br. J. Cancer. 2018;118(8):1130-41.

2. World Cancer Research Fund/American Institute for Cancer Research. Diet, Nutrition, Physical Activity and Cancer: a Global Perspective. Continuous Update Project Expert Report. 2018. [Internet] Available at: dietandcancerreport.org. Accessed 20 Jan 2020.

3. Thomas, R, Davies, N. Lifestyle during and after cancer treatment. Clin. Oncol. 2007;19(8): 616-627.

4. Chan DS, Vieira AR, Aune D, Bandera EV, Greenwood DC, McTiernan A et al. Body mass index and survival in women with breast cancer—systematic literature review and meta-analysis of 82 follow-up studies. Ann. Oncol. 2014 Oct 1;25(10):1901-14.

5. Speck, R.M., Courneya, K.S., Mâsse, L.C., Duval, S. & Schmitz, K.H. An update of controlled physical activity trials in cancer survivors: a systematic review and meta-analysis. J Cancer Surviv. 2010;4 (2):87-100.

6. Pierce JP, Stefanick ML, Flatt SW, Natarajan L, Sternfeld B, Madlensky L et al. Greater survival after breast cancer in physically active women with high vegetable-fruit intake regardless of obesity. J. Clin. Oncol. 2007;25(17):2345–2351.

7. World Cancer Research Fund/American Institute for Cancer Research. Continuous Update Project Expert Report. 2018. Survivors of breast and other cancers. [Internet] Available at: https://www.wcrf.org/sites/default/files/Breast-cancer-survivors-report.pdf. Accessed: 16 Jan 2020. Accessed 2 April 2020.

8. Meyerhardt JA, Niedzwiecki D, Hollis D, Saltz LB, Hu FB, Mayer RJ et al. Association of dietary patterns with cancer recurrence and survival in patients with stage III colon cancer. Jama. 2007;298(7):754-64.

9.Tollosa DN, Tavener M, Hure A, James EL. Adherence to multiple health behaviours in cancer survivors: a systematic review and meta-analysis. J Cancer Surviv. 2019;13(3):327-43.

For peer review only - http://bmjopen.bmj.com/site/about/guidelines.xhtml

10. Pugh G, Hough R, Gravestock H, Fisher A. The health behaviour status of teenage and young adult cancer patients and survivors in the United Kingdom. Support. Care Cancer. 2020;28(2):767-77

11. Corbett T, Cheetham T, Müller AM, Slodkowska-Barabasz J, Wilde L, Krusche A et al. Exploring cancer survivors' views of health behaviour change: "Where do you start, where do you stop with everything?". Psycho-oncology. 2018;27(7):1816-24.

12. Jones, L.W., Courneya, K.S., Fairey, A.S., Mackey, J.R. Effects of an oncologist's recommendation to exercise on self-reported exercise behavior in newly diagnosed breast cancer survivors: a single-blind, randomized controlled trial. Ann. Behav. Med. 2004;28(2):105-13

13. Beeken RJ, Williams K, Wardle J, Croker H. "What about diet?" A qualitative study of cancer survivors' views on diet and cancer and their sources of information. Eur. J. Cancer Care. 2016;25(5):774-83.

14. Daley AJ, Bowden SJ, Rea DW, Billingham L, Carmicheal AR. What advice are oncologists and surgeons in the United Kingdom giving to breast cancer patients about physical activity? Int. J. Behav. Nutr. Phys. Act. 2008;5(1):46.

15. Pattinson L, Jessop A. The delivery of health improvement information during radiotherapy treatment: a survey of UK therapy radiographers. J Radiother Pract. 2016; 15 (2): 114–130.

16. Williams K, Beeken RJ, Fisher A, Wardle J. Health professionals' provision of lifestyle advice in the oncology context in the United Kingdom. Eur J Cancer Care. 2015;24(4):522-30.

17. Koutoukidis DA, Lopes S, Fisher A, Williams K, Croker H, Beeken RJ. Lifestyle advice to cancer survivors: a qualitative study on the perspectives of health professionals. BMJ open. 2018;8(3).

18. Cantwell M, Walsh D, Furlong B, Moyna N, McCaffrey N, Boran L et al. Healthcare professionals' knowledge and practice of physical activity promotion in cancer care: Challenges and solutions. Eur J Cancer Care. 2018;27(2):12795.

19. Conlon K, Pattinson L, Hutton D. Attitudes of oncology healthcare practitioners towards smoking cessation: A systematic review of the facilitators, barriers and recommendations for delivery of advice and support to cancer patients. Radiography. 2017;23: 256-263

For peer review only - http://bmjopen.bmj.com/site/about/guidelines.xhtml

20. Sutton E, Hackshaw-McGeagh LE, Aning J, Bahl A, Koupparis A, Persad R et al. The provision of dietary and physical activity advice for men diagnosed with prostate cancer: a qualitative study of the experiences and views of health care professionals, patients and partners. Cancer Causes Control. 20171;28(4):319-29.

21. Spellman C, Craike M, Livingston PM. Knowledge, attitudes and practices of clinicians in promoting physical activity to prostate cancer survivors. Health Educ J. 2013:1– 10. 34.

22. Cancer Research UK. Vision for radiotherapy. 2014.

23. Halkett GK, Kristjanson LJ. Patients' perspectives on the role of radiation therapists. Patient Education and Counselling. 2007;69(1):76-83

24. Society and College of Radiographers (SCoR). The College of Radiographers Research Priorities for the Radiographic Profession. 2017

25. Chief Allied Heath Professions Officer's Team. Allied Health Professions into Action. Using Allied Health Professionals to transform health, care and wellbeing. 2017. NHS England

26. Allied Health Professions Federation. UK Allied Health Professions Public Health Strategic Framework 2019-2024. 2019. [Internet] Available at: http://www.ahpf.org.uk/files/UK%20AHP%20Public%20Health%20Strategic%20Framework%202019-2024.pdf. Accessed 17 Jan 2020.

27. NHS. An Implementation Guide and Toolkit for Making Every Contact Count: Using every opportunity to achieve health and wellbeing. 2016

28. Charlesworth L, Hutton D, Hussain H. Therapeutic Radiographers' perceptions of the barriers and enablers to effective smoking cessation support. Radiography. 2019;25(2):121-8.

29. Boaz, A, Baeza, J, Fraser, A. Effective implementation of research into practice: an overview of systematic reviews of the health literature. BMC research notes 2011; 4 (1): 212.

30. Gill P, Stewart K, Treasure E, and Chadwick B. Methods of data collection in qualitative research: interviews and focus groups. Br Dent J. 2008; 204(6):291-5.

For peer review only - http://bmjopen.bmj.com/site/about/guidelines.xhtml

31. Guest G, Bunce A, Johnson L. How many interviews are enough? An experiment with data saturation and variability. Field methods. 2006;18(1):59-82.

32. Saunders B, Sim J, Kingstone T, Baker S, Waterfield J, Bartlam B et al. Saturation in qualitative research: exploring its conceptualization and operationalization. Quality quantity. 2018;52(4):1893-907.

33. Gale NK, Heath G, Cameron E, Rashid S, Redwood S. Using the framework method for the analysis of qualitative data in multi-disciplinary health research. BMC medical research methodology. 2013;13(1):117.

34. Tong A, Sainsbury P, Craig J. Consolidated criteria for reporting qualitative research (COREQ): a 32-item checklist for interviews and focus groups. International journal for quality in health care. 2007;19(6):349-57.

35. Society of Radiographer. Radiotherapy radiographic workforce UK census 2018. [Internet] Available at https://www.sor.org/learning/document-library/radiotherapy-radiographic-workforce-uk-census-2018. Accessed 1 March 2020.

36. Williamson K, Williams KJ. Organisational justice, trust and perceptions of fairness in the implementation of agenda for change. Radiography. 2011;17(1):61-6.

37. McClinchy J, Williams J, Gordon L, Cairns M, Fairey G. Dietary Advice and Collaborative Working: Do Pharmacists and Allied Health Professionals Other Than Dietitians Have a Role? InHealthcare. 2015; 3(1):64-77. Multidisciplinary Digital Publishing Institute.

38. Scarborough P, Bhatnagar P, Wickramasinghe KK, Allender S, Foster C, Rayner M. The economic burden of ill health due to diet, physical inactivity, smoking, alcohol and obesity in the UK: an update to 2006–07 NHS costs. J. Public Health. 2011;33(4):527-35.

39. Thompson RL, Summerbell CD, Hooper L, Higgins JP, Little P, Talbot D et al. Dietary advice given by a dietitian versus other health professional or self-help resources to reduce blood cholesterol. Cochrane Database of Systematic Reviews. 2003(3).

40. The NHS. The NHS long term plan. 2019

For peer review only - http://bmjopen.bmj.com/site/about/guidelines.xhtml

41. Independent Cancer Taskforce. Achieving world-class cancer outcomes a strategy for England 2015-2020. 2015

42. Ruiz JG, Mintzer MJ, Leipzig RM. The impact of e-learning in medical education. Academic medicine. 2006; 81(3):207-12.

43. Childs S, Blenkinsopp E, Hall A, Walton G. Effective e-learning for health professionals and students—barriers and their solutions. A systematic review of the literature—findings from the HeXL project. Health Information & Libraries Journal. 2005;22:20-32.

44. Cook DA, Levinson AJ, Garside S, Dupras DM, Erwin PJ, Montori VM. Internet-based learning in the health professions: a meta-analysis. Jama. 2008;300(10):1181-96.

45. Gray-Burrows KA, Willis TA, Foy R, Rathfelder M, Bland P, Chin A, Hodgson S, Ibegbuna G, Prestwich G, Samuel K, Wood L. Role of patient and public involvement in implementation research: a consensus study. BMJ quality & safety. 2018;27(10):858-64.

46. Avery KN, Donovan JL, Horwood J, Neal DE, Hamdy FC, Parker C et al. The importance of dietary change for men diagnosed with and at risk of prostate cancer: a multi-centre interview study with men, their partners and health professionals. BMC family practice. 2014;15(1):81.

47. Denmark et al. 2000) Demark-Wahnefried W, Peterson B, McBride C, Lipkus I, Clipp E. Current health behaviors and readiness to pursue life-style changes among men and women diagnosed with early stage prostate and breast carcinomas. Cancer. 2000;88(3):674–84

48. Murphy, J.L. & Girot, E.A. The importance of nutrition, diet and lifestyle advice for cancer survivors–the role of nursing staff and interprofessional workers. J Clin Nurs. 2013;22(11-12):1539-49.

49. Lahart, I.M., Metsios, G.S., Nevill, A.M., Carmichael, A.R. Physical activity, risk of death and recurrence in breast cancer survivors: A systematic review and meta-analysis of epidemiological studies. Acta Oncol. 2015;54(5):635-54.

50. Van Blarigan EL, Meyerhardt JA. Role of physical activity and diet after colorectal cancer diagnosis. Journal of Clinical Oncology. 2015 Jun 1;33(16):1825.

For peer review only - http://bmjopen.bmj.com/site/about/guidelines.xhtml

51. Macmillan Cancer Support. [2015] The burden of cancer and other long-term health conditions. Available at: https://www.macmillan.org.uk/documents/press/cancerandotherlong-termconditions.pdf. Accessed 27th Match 2020.

52. Cummings A, Grimmett C, Calman L, Patel M, Permyakova NV, Winter J, et al. Comorbidities are associated with poorer quality of life and functioning and worse symptoms in the 5 years following colorectal cancer surgery: results from the ColoREctal well-being (CREW) cohort study. Psychooncology. 2018;27(10):2427–35

53. Marmot M, Atinmo T, Byers T, Chen J, Hirohata T, Jackson A et al. Food, nutrition, physical activity, and the prevention of cancer: a global perspective. Office for national statistics. 2019. Cancer registration statistics, England: 2017.

54. National Cancer Registration & Analysis Service and Cancer Research UK: Chemotherapy, Radiotherapy and Tumour Resections in England: 2013-2014 workbook. London: NCRAS; 2017.

55. Council of Deans of Health. (2017). Guidance: Public Health Content within the Pre-Registration Curricula for Allied Health Professions. [Internet] Available at: https://councilofdeans.org.uk/wp-content/uploads/2017/09/Public-Health-Content-within-the-Pre-Registration-Curricula-for-Allied-Health-Professions.pdf. Accessed 20 January 2020.

For peer review only - http://bmjopen.bmj.com/site/about/guidelines.xhtml

Supplementary material 1: Interview topic guide

Topic	Question	Prompt
**Introduction**	Brief introductions; get verbal consent to tape-record the interview; remind the aims of study, check length of interview/audio-recorded.	
**Health promotion beliefs**	What are your views on the role of health behaviours in those with a diagnosis of cancer?<br><br>Do you think delivering advice on improving health behaviours is part of your role?	Breast/prostate/colorectal/head & neck cancers<br><br>Healthy eating/ exercise/ alcohol/ smoking / weight management
**Provision of advice on healthy behaviours**	Can you tell me about your experiences of providing health behaviour advice to cancer patients?<br><br>Are there some health behaviours you recommend more than others?<br><br>Why do you think that is?<br><br>Are there some patient groups that you provide health behaviour advice to more than others?<br><br>Why do you think that is?	Healthy eating/ exercise/ alcohol/ smoking / weight management
**Barriers to health advice delivery**	On the survey we asked you about a number of barriers limiting you in providing health behaviour advice to your patients. You mentioned X Y Z<br><br>Can you tell me more about these barriers?<br><br>Can you think of any other barriers within your role and work environment stopping you from giving lifestyle and health promotion advice to patients?	Why do you think that is?
**Addressing barriers**	What support do you think would be most helpful in enabling therapeutic radiographers to deliver health behaviour advice to patients?	
**Training preferences**	How helpful do you think an online training course specifically for therapeutic radiographers on delivering health behaviour advice to cancer patients would be?<br><br>How helpful do you think a face to face training course specifically for therapeutic radiographers on delivering health behaviour advice to cancer patients would be?<br><br>If you were provided with a training course (online or face to face) what topics would you like delivered?	Why do you think this is?
**Finish**	Thank you for your time. Do you have anything else you would like to add?	

For peer review only

COREQ (COnsolidated criteria for REporting Qualitative research) Checklist

A checklist of items that should be included in reports of qualitative research. You must report the page number in your manuscript where you consider each of the items listed in this checklist. If you have not included this information, either revise your manuscript accordingly before submitting or note N/A.

Topic	Item No.	Guide Questions/Description	Reported on Page No.
**Domain 1: Research team and reflexivity**			
Personal characteristics			
Interviewer/facilitator	1	Which author/s conducted the interview or focus group?	
Credentials	2	What were the researcher's credentials? E.g. PhD, MD	
Occupation	3	What was their occupation at the time of the study?	
Gender	4	Was the researcher male or female?	
Experience and training	5	What experience or training did the researcher have?	
Relationship with participants			
Relationship established	6	Was a relationship established prior to study commencement?	
Participant knowledge of the interviewer	7	What did the participants know about the researcher? e.g. personal goals, reasons for doing the research	
Interviewer characteristics	8	What characteristics were reported about the inter viewer/facilitator? e.g. Bias, assumptions, reasons and interests in the research topic	
**Domain 2: Study design**			
Theoretical framework			
Methodological orientation and Theory	9	What methodological orientation was stated to underpin the study? e.g. grounded theory, discourse analysis, ethnography, phenomenology, content analysis	
Participant selection			
Sampling	10	How were participants selected? e.g. purposive, convenience, consecutive, snowball	
Method of approach	11	How were participants approached? e.g. face-to-face, telephone, mail, email	
Sample size	12	How many participants were in the study?	
Non-participation	13	How many people refused to participate or dropped out? Reasons?	
Setting			
Setting of data collection	14	Where was the data collected? e.g. home, clinic, workplace	
Presence of non-participants	15	Was anyone else present besides the participants and researchers?	
Description of sample	16	What are the important characteristics of the sample? e.g. demographic data, date	
Data collection			
Interview guide	17	Were questions, prompts, guides provided by the authors? Was it pilot tested?	
Repeat interviews	18	Were repeat inter views carried out? If yes, how many?	
Audio/visual recording	19	Did the research use audio or visual recording to collect the data?	
Field notes	20	Were field notes made during and/or after the inter view or focus group?	
Duration	21	What was the duration of the inter views or focus group?	
Data saturation	22	Was data saturation discussed?	
Transcripts returned	23	Were transcripts returned to participants for comment and/or	

For peer review only

Topic	Item No.	Guide Questions/Description	Reported on Page No.
		correction?	
**Domain 3: analysis and findings**			
Data analysis			
Number of data coders	24	How many data coders coded the data?	
Description of the coding tree	25	Did authors provide a description of the coding tree?	
Derivation of themes	26	Were themes identified in advance or derived from the data?	
Software	27	What software, if applicable, was used to manage the data?	
Participant checking	28	Did participants provide feedback on the findings?	
Reporting			
Quotations presented	29	Were participant quotations presented to illustrate the themes/findings? Was each quotation identified? e.g. participant number	
Data and findings consistent	30	Was there consistency between the data presented and the findings?	
Clarity of major themes	31	Were major themes clearly presented in the findings?	
Clarity of minor themes	32	Is there a description of diverse cases or discussion of minor themes?	

Developed from: Tong A, Sainsbury P, Craig J. Consolidated criteria for reporting qualitative research (COREQ): a 32-item checklist for interviews and focus groups. *International Journal for Quality in Health Care*. 2007. Volume 19, Number 6: pp. 349 – 357

Once you have completed this checklist, please save a copy and upload it as part of your submission. DO NOT include this checklist as part of the main manuscript document. It must be uploaded as a separate file.

For peer review only

BMJ Open

Therapeutic radiographers' delivery of health behaviour change advice to those living with and beyond cancer: a qualitative study.

Journal:	*BMJ Open*
Manuscript ID	bmjopen-2020-039909.R2
Article Type:	Original research
Date Submitted by the Author:	11-Jun-2020
Complete List of Authors:	Pallin, Nickola; University College London, Behavioural Science and Health; London South Bank University, Department of Allied Health Sciences<br>Beeken, Rebecca; University College London, Behavioural Science and Health; University of Leeds Leeds Institute of Health Sciences<br>Pritchard-Jones, Kathy; London Cancer<br>Charlesworth, Laura; University of Lincoln<br>Woznitza, Nicholas; Homerton University Hospital NHS Foundation Trust, Radiology; Canterbury Christ Church University, Canterbury Christ Church University<br>Fisher, Abigail; University College London, Behavioural Science and Health
**Primary Subject Heading**:	Oncology
Secondary Subject Heading:	Public health
Keywords:	RADIOTHERAPY, MEDICAL EDUCATION & TRAINING, Adult oncology < ONCOLOGY, QUALITATIVE RESEARCH

SCHOLARONE™
Manuscripts

I, the Submitting Author has the right to grant and does grant on behalf of all authors of the Work (as defined in the below author licence), an exclusive licence and/or a non-exclusive licence for contributions from authors who are: i) UK Crown employees; ii) where BMJ has agreed a CC-BY licence shall apply, and/or iii) in accordance with the terms applicable for US Federal Government officers or employees acting as part of their official duties; on a worldwide, perpetual, irrevocable, royalty-free basis to BMJ Publishing Group Ltd ("BMJ") its licensees and where the relevant Journal is co-owned by BMJ to the co-owners of the Journal, to publish the Work in this journal and any other BMJ products and to exploit all rights, as set out in our licence.

The Submitting Author accepts and understands that any supply made under these terms is made by BMJ to the Submitting Author unless you are acting as an employee on behalf of your employer or a postgraduate student of an affiliated institution which is paying any applicable article publishing charge ("APC") for Open Access articles. Where the Submitting Author wishes to make the Work available on an Open Access basis (and intends to pay the relevant APC), the terms of reuse of such Open Access shall be governed by a Creative Commons licence – details of these licences and which Creative Commons licence will apply to this Work are set out in our licence referred to above.

Other than as permitted in any relevant BMJ Author's Self Archiving Policies, I confirm this Work has not been accepted for publication elsewhere, is not being considered for publication elsewhere and does not duplicate material already published. I confirm all authors consent to publication of this Work and authorise the granting of this licence.

For peer review only

Title: Therapeutic radiographers' delivery of health behaviour change advice to those living with and beyond cancer: a qualitative study.

Authors

Pallin N D [1, 2] Beeken R J [1,3] Pritchard Jones K [4] Charlesworth L[5] Woznitza N[6] Fisher A[1]

1. Department of Behavioural Science and Health, University College London, London, UK
2. Department of Allied Health Sciences, London South Bank University, London, UK
3. Leeds Institute of Health Sciences, University of Leeds, Leeds, UK
4. London Cancer, London, UK
5. College of Social Science, University of Lincoln, Lincolnshire, UK
6. Radiology Department, Homerton University Hospital, London, UK

Nickola Pallin email: pallinn@lsbu.ac.uk

Dr Rebecca Beeken email: R.Beeken@leeds.ac.uk

Prof Kathy Pritchard Jones: k.pritchard-jones@ucl.ac.uk

Laura Charlesworth: LCharlesworth@lincoln.ac.uk

Dr Nick Woznitza: Nicholas.woznitza@nhs.net

Dr Abi Fisher email: Abigail.fisher@ucl.ac.uk

Present address of corresponding author: Nickola Pallin

School of Health and Social Care

Department of Allied Health Sciences

London South Bank University

103 Borough Road

London

SE1 0AA

Tel: 0207815674

Email: pallinn@lsbu.ac.uk

Note: This work was undertaken in the Department of Behavioural Science and Health, University College London, London, UK. The lead author was based there as a pre-doctoral research fellow.

For peer review only - http://bmjopen.bmj.com/site/about/guidelines.xhtml

Abstract

Objectives Therapeutic radiographers (TRs) are well placed to deliver health behaviour change advice to those living with and beyond cancer (LWBC). However, there is limited research on the opinions of TRs around delivering such advice to those LWBC. This study aimed to explore TRs' practices and facilitators in delivering advice on physical activity, healthy eating, alcohol intake, smoking and weight management

Setting and Participants Fifteen UK-based TRs took part in a telephone interview using a semi-structured interview guide. Data was analysed using the framework analysis method.

Results Emergent themes highlighted that TRs are mainly aware of the benefits of healthy behaviours in managing radiotherapy treatment related side effects, with advice provision lowest for healthy eating and physical activity. Participants identified themselves as well placed to deliver advice on improving behaviours to those LWBC, however reported a lack of knowledge as a limiting factor to doing so. The TRs reported training and knowledge as key facilitators to the delivery of advice, with a preference for online training.

Conclusions There is a need for education resources, clear referral pathways and in particular training for TRs on delivering physical activity and healthy eating advice to those LWBC.

For peer review only

For peer review only - http://bmjopen.bmj.com/site/about/guidelines.xhtml

Strengths and limitations of this study

- This study provides insight in therapeutic radiographers' views on all key modifiable health behaviours for those living with and beyond cancer.
- The participants worked in different radiotherapy departments, offering insight into the practices among therapeutic radiographers in the delivery of healthy behaviour advice from a wide range of hospitals.
- Whilst data saturation was reached, the sample size was small and therefore the findings may not be representative of the views of the wider therapeutic radiography workforce.
- The response rate was low (20.8%), therefore the participants might be more interested in the role of health behaviours in cancer survivorship, which might bias the responses towards a positive view on the role of therapeutic radiographers in delivering advice within their role.

Keywords: Therapeutic Radiographer, cancer, health behaviours, healthy eating, physical activity, lifestyle, behaviour change

For peer review only - http://bmjopen.bmj.com/site/about/guidelines.xhtml

Introduction

It is estimated that 40% of cancer cases are linked to unhealthy behaviours [1]. Based on evidence from systematic literature reviews and meta analyses, the World Cancer Research Fund (WCRF) recommend that individuals are physically active, limit consumption of energy dense foods, salty foods, red meat and avoid processed meat, eat more plant foods, maintain a healthy weight, limit alcoholic drinks and avoid tobacco to reduce their risk of cancer [2]. Those living with and beyond cancer (LWBC) are also advised to follow these guidelines, due to increasing evidence that healthy behaviours may improve physical and psychosocial outcomes after a cancer diagnosis [2 - 8].

Despite the potential benefits of healthy behaviours, few people LWBC are meeting the WCRF recommended health behaviour recommendations [9, 10]. Those LWBC report one key reason for not adopting healthier lifestyle behaviours is lack of advice and support from their healthcare team [11]. Healthcare professionals (HCPs) are well placed to bring about positive health behaviour changes among cancer patients [12]. A trial of brief advice among breast cancer survivors showed that a simple physical activity recommendation from a HCP doubled the percentage meeting national exercise guidelines [12]. Despite this, research to date among both HCPs and those LWBC consistently shows that few oncology HCPs offer guidance to oncology patients on healthy lifestyle behaviours [13 – 21].

Reported barriers among HCPs in providing health behaviour advice for those LWBC, include believing that giving advice was not part of their role, lack of time with patients, lack of referral programmes, lack of resources such as education leaflets for those LWBC, and lack of knowledge regarding guidelines and research findings [16 – 21]. A recent qualitative study with 21 oncology HCPs identified that advice on health behaviours provided to those LWBC focused on general health and controlling side effects, with few HCPs advising on health behaviours in the context of improving survival outcomes [20]. Whilst these studies provide useful insight into the practices and barriers among oncology HCPs the participants within these studies were primarily oncologists and nurses and

For peer review only - http://bmjopen.bmj.com/site/about/guidelines.xhtml

focused on the provision of physical activity and weight management advice. There is limited research on the opinions of therapeutic radiographers (TRs) in delivering advice on health behaviours to those LWBC. Despite at least 50% of cancer patients receiving radiotherapy as part of their cancer treatment [22].

TRs are the only health professionals qualified to deliver radiotherapy and play a central role in supporting cancer patients [23]. In the UK, the College of Radiographers recognise the importance of TRs in providing health behaviour advice to improve patient outcomes [24]. TRs are also seen as an integral part of the health force in driving improvements in wellbeing as outlined in the 2017 publication of "AHPs into Action, using Allied Health Professionals to transform health, care and wellbeing" which states that radiographers are key to implementing a preventative healthcare approach and that their expertise should be used to design and deliver health interventions [25]. TRs are ideally placed to deliver health behaviour advice, particularly through Making Every Contact Count (MECC) [26,27.] MECC is a strategy whereby health professionals use every appropriate opportunity and interaction with patients to promote healthy behaviours and signpost to relevant healthcare services using an 'Ask, Advise, Act' framework [27]. MECC fits extremely well within the TRs' role in which patient education is a key part of radiotherapy practice with TRs providing care to the same patient every day, often over a number of weeks [23]. TRs therefore have the potential to make significant contributions in supporting positive health behaviour changes among those LWBC.

However, despite these opportunities one survey in the UK among 102 TRs identified that TRs rarely advise patients on the key modifiable health behaviours including smoking, alcohol, healthy eating and exercise [15]. The findings also showed lack of knowledge and training as barriers among TRs in delivering advice on these topics [15]. Similarly, focus group interviews with 38 TRs identified that lack of knowledge and training were barriers to the provision of smoking cessation advice [28].

For peer review only - http://bmjopen.bmj.com/site/about/guidelines.xhtml

Challenges remain in translating behaviour change interventions into existing care pathways and practices in a way that is appropriate for use by health professionals [29]. Understanding TRs' practices, and what support they need in delivering advice on the topics of physical activity, healthy eating, alcohol intake, smoking and weight management could inform the development of interventions that will enable TRs' in delivering advice on improving health behaviours to those LWBC. Qualitative research is appropriate for exploring the beliefs, experiences and motivations of individuals on specific matters and allow for more information and clarification [30]. Limited qualitative data exists on TRs' practices and views on delivering advice on these health behaviour topics. This study therefore aimed to address this and through a qualitative methodology explore TRs' practices in delivering health behaviour advice, in addition to exploring the facilitators in delivering such advice. Preferences regarding training on delivering this advice were also explored.

Methods

Participants and Recruitment

Participants were TRs working in the UK in a clinical role. They had provided their contact details on a previous online survey investigating TR's practices in delivering health behaviour advice agreeing to be invited for a follow up telephone interview. An email was sent with an information sheet explaining the research and inviting these TRs. Those who agreed to take part signed a consent form prior to the telephone interview. Ethical approval was granted by the University College London Research Ethics Committee, reference 12945/001.

Data collection

Semi-structured individual telephone interviews were carried out between April and May 2019 by a lecturer in therapeutic radiography with an MSc who had completed qualitative interviewing as part of their training (NP). The interviewer had no previous relationship with the study participants. The

For peer review only - http://bmjopen.bmj.com/site/about/guidelines.xhtml

topic guide (supplementary material 1) was based on the guide used within a previous study [17] which explored oncology HCPs views on the provision of lifestyle advice to cancer patients. This guide was adapted for use among TRs, with additional questions added to assess preferences for training on delivering advice. The topic guide was piloted with two participants to check for comprehension of the questions. This data was included in the analysis because no substantial changes were required. The interviews lasted approximately 30 min (range: 20 – 40 min) and were audio-recorded, anonymised and transcribed verbatim. The transcripts were verified by NP against each recording to confirm accuracy. The aim was to carry out interviews until data saturation was reached. It was anticipated that ten participants would be required to reach data saturation because it was a homogeneous group [31]. After ten interviews were carried out, they were transcribed verbatim. Following familiarisation with the data NP generated the initial codes and it appeared that saturation was reached after ten interviews as no new codes occurred in the tenth interview [32]. A further five interviews were carried out to confirm this.

Patient and public involvement

Patient input was not used in the design of the research methods. However, the topic guide was piloted with TRs in the academic setting. Additionally, the topic guide was piloted with two participants and these were included in the analysis.

Analysis

The interview transcripts were analysed using the framework analysis method [33]. This method was chosen because it is an appropriate method for analysing homogenous data and semi-structured interview transcripts, it is also appropriate when using inductive qualitative analysis [33]. A random selection of transcripts (n = 3) were independently coded by AF to check for reliability. The researchers NP, AF and RJB met and agreed on a final coding list in an iterative process (AF and RJB are both experienced qualitative researchers and health psychologists). These agreed set of codes formed the

For peer review only - http://bmjopen.bmj.com/site/about/guidelines.xhtml

analytical framework which was then applied to all of the transcripts and the data summarised in a matrix using Microsoft excel. Themes were generated by reviewing the matrix connecting the data between the participants and the codes. The completed consolidated criteria for reporting qualitative research checklist is available in the supplementary material 2 [34]. Themes are presented in the results with supporting quotes and the participants identifier (Table 1).

Table 1: Participant identifier and demographic characteristics

Participant Identifier	Demographic characteristics	
	**Gender**	**Professional Grade**
TR 1	Female	Band 6
TR 2	Female	Band 7
TR 3	Female	Band 7
TR 4	Female	Band 7
TR 5	Female	Band 7
TR 6	Female	Band 7
TR 7	Female	Band 7
TR 8	Male	Band 8
TR 9	Female	Band 7
TR 10	Female	Band 7
TR 11	Female	Band 6
TR 12	Female	Band 7
TR 13	Male	Band 5
TR 14	Female	Band 7
TR 15	Male	Band 8

TR: Therapeutic Radiographer

Results

Participants

The radiotherapy radiography workforce census in the UK only reports the workforce's professional grade, and no other demographics [35]. In the UK, TRs' level of professional skills and knowledge are categorised by agenda for change (AfC) professional band grades 5 to 8 [36]. Therefore, in this study, the participants gender and professional grade were collected; no other demographic information

For peer review only - http://bmjopen.bmj.com/site/about/guidelines.xhtml

was collected (Table 1). The response rate to taking part in the interview was 20.8%. Seventy-two TRs were emailed and invited to take part in an interview, 15 returned consent forms and completed the telephone interview. Fifteen interviews were conducted with 12 women and 3 men. The participants came from all regions of the UK, including England, Wales, Scotland and Northern Ireland.

Themes

Five main themes were identified: (1) TRs provide behaviour change advice to manage radiotherapy related side effects (2) TRs make judgements about when it is appropriate to deliver health behaviour advice (3) Knowledge and training are key facilitators in the delivery of health behaviour advice (4) TRs feel patients undergoing radiotherapy treatment seek guidance on health behaviours (5) TRs identify themselves as well placed to give health behaviour advice to patients.

TRs provide behaviour change advice to manage radiotherapy related side effects

Most respondents reported that they only provided advice on health behaviours that they believed would minimise radiotherapy related side effects. This meant smoking cessation and alcohol intake were the two health behaviours TRs mainly advised on.

"With head and neck patients, we give advice, particularly on smoking and drinking, obviously get worse side effects" (TR 6).

"The only thing we do generally say is about drinking plenty of fluids, avoiding alcohol. But that's more to do with prostate side effects, bladder reactions, and reducing gas" (TR 14).

"Radiographer are comfortable talking about alcohol when it comes to managing side effects" (TR 12).

No TRs reported advising patients on healthy eating. Some TRs mentioned advising patients on dietary

For peer review only - http://bmjopen.bmj.com/site/about/guidelines.xhtml

intake but this is to patients who are at risk of losing weight, for side effect management and potential impact on accuracy of radiotherapy treatment delivery.

"Healthy eating, I don't tend to discuss too much. A lot of patients have difficulty eating and we are encouraging maintaining weight while on treatment" (TR 5).

"I'm not very sure if healthy eating is important. Any patients where we're treating, lower GI or pelvis, we would advise them to avoid very high-fibre foods, spicy foods, that might make them have very loose bowels, but other than that we say more or less keep on your same diet. We wouldn't generally discuss a healthy diet as a standard for all patients. No" (TR 15).

Some TRs mentioned they advise patients to be physically active, however this was only in the context of managing radiotherapy and cancer related fatigue.

"So exercise is one of my main ones that I focus on with all patients, particularly to help with their fatigue" (TR 9).

"Exercise, I say that's its quite beneficial to help with fatigue" (TR 12).

"I guess when we have patients come in, fatigue is one of the side effects, so we encourage our patients to remain active" (TR 15).

TRs make judgements about when it is appropriate to deliver health behaviour advice

TRs explained only discussing health behaviours, particularly smoking and alcohol with patients if there were evident indications of a problem. TRs also often reported making a judgment of whether appropriate to advise a patient on a particular health behaviour.

For peer review only - http://bmjopen.bmj.com/site/about/guidelines.xhtml

"So, quite often you can tell if a patient is a smoker, you can smell it, or you can tell by their skin" (TR 11).

"I tend to give advice when you make a judgement of when it's appropriate, an example might be if a patient smell of smoke" (TR 12).

"Had patients come in and will smell of alcohol and at that time I'll say to the patient that it can exacerbate side effects" (TR 15).

This meant TRs did not provide advice on health behaviours to every patient.

"But for those patients where it's not clearly going to benefit them to stop drinking, you would just mention it very briefly. Not every patient will have that information" (TR 5).

Knowledge and training are keys facilitator in the delivery of health behaviour advice

Delivery of advice matched by knowledge

The reported delivery of advice on health behaviours appeared to be matched by knowledge of the benefits among those LWBC.

One participant explained how he only appreciated the importance of PA in cancer survivorship after attending a talk and being made aware of the evidence.

"My experience of appreciating the role exercise was from attending a talk. I suppose it was really just highlighting in the studies the benefits obviously of a healthy lifestyle and introducing physical activity

For peer review only - http://bmjopen.bmj.com/site/about/guidelines.xhtml

for patients on treatment" (TR 15).

Healthy eating was a topic the participants felt particularly unqualified to deliver advice on, and reported lack of knowledge as a barrier to the delivery of advice on healthy eating.

"It's a difficult one, diet, I think. It's more a knowledge thing. If you don't have the knowledge about what you can and can't say, you're just not going to approach the subject" (TR 12).

A need for continuous post graduate online training

All interviewed said they would welcome post graduate training on delivering health behaviour advice. The majority expressed a preference for online training to help overcome the barrier of limited time among TRs to attend training.

"Online, you're not having to take time out of clinical practice, online is more accessible" (TR 6)

Participants also mentioned that online training allows for yearly updates and continuous professional development (CPD).

"I think it'd be good (online training) because you can do it in your own time. Because I think that's sometimes the problem. You have this training once and then maybe it never gets brought up again. So it would be quite handy to have something small, every year, alongside all your other mandatory training" (TR 12).

Participants did acknowledge that face to face training allows for further questioning that's not possible with online training.

For peer review only - http://bmjopen.bmj.com/site/about/guidelines.xhtml

"I think one to one training, because you can ask questions that may not be covered within the online training" (TR 1).

To overcome of the barrier of not all staff being able attend face to face training participants suggested it would be useful to train some TRs through face to face methods that they could then cascade to other TRs within the radiotherapy department.

"Maybe some face-to-face with some staff, that they could cascade down, might be useful as well" (TR 4).

A need for training in the undergraduate setting

It was also suggested to incorporate training on delivering lifestyle advice into the undergraduate education program.

"Certainly, get it into the undergraduate course to start with, making them aware it is part of the role" (TR 9).

"It's still not something that I can say was primarily covered in the undergraduates' training about the benefits of healthy lifestyle, you know, there's no real formal education that I can see" (TR 15).

TRs reported knowledge of resources and referral pathways as facilitators in the delivery of advice

Participants also felt knowledge of how to refer patients onto further support would enable them to have conversations on improving health behaviours. With some TRs reporting that lack of knowledge of resources and referral pathways are barriers to initiating a conversation on behaviour change.

"There needs to be more information available to professionals of where exactly you can refer patients

For peer review only - http://bmjopen.bmj.com/site/about/guidelines.xhtml

to, whether that be website, whether that be an app" (TR 13).

"That's the only reason why they (therapeutic radiographers) don't want to open these conversations up, because they don't know where to go with it or how to refer on" (TR 9).

They also acknowledge that in the short time they have with patients if they had a resource then would be more inclined to advise.

"Having something on a piece of paper, education and having the resources, if you can do it in two minutes you should be able slip that in" (TR 2).

"You don't always have that information at hand, so if it was readily available, I think we'd give out a bit more (health behaviour information) if it was just the case of pointing them in the right direction that would be a quick and easy thing to do" (TR 3).

The benefit of incorporating patients' perspective into training

Participants also mentioned that getting patients' perspectives on receiving advice on improving health behaviours should be incorporated into training.

"I think that would be better coming from the patients themselves, rather than just feedback from what journals, and other literature says" (TR 7).

"If there'd even be patients that would be willing to maybe just even be involved with staff training" (TR 3).

TRs feel patients undergoing radiotherapy treatment seek guidance on health behaviours.

For peer review only - http://bmjopen.bmj.com/site/about/guidelines.xhtml

Many of the TRs also described that patients often ask them for guidance around health behaviour changes, particularly on diet and exercise. This shows that patients see TRs as credible sources of information on health behaviours.

"We are getting asked the question more and more about weight loss, healthy living, wanting to exercise more" (TR 4).

"It is quite a common thing to be asked at the end of treatment, not so much the smoking and alcohol, I have to say, but diet and exercise is certainly something that people commonly ask" (TR 3).

TRs identify themselves as well placed to give health behaviour advice to patients.

TRs acknowledged that they are a consistent healthcare member for patients undergoing radiotherapy and have many opportunities to deliver lifestyle advice. Therefore, TRs recognised that they are well placed to deliver health behaviour advice to patients.

"We're in a unique position because we do see the same patient day after day and you do kind of start to develop a relationship with them" (TR 10).

"I think we're well placed to help influence patients' behaviours and it' something we should be seen to encourage and report" (TR 7).

"We're in the best position where we see the patients, for a number of weeks every day to encourage any changes" (TR 8).

From the interviews it appeared that many patients undergoing radiotherapy, excluding those at risk of malnutrition or significant weight loss, are primarily reviewed and assessed by TRs. This highlights

For peer review only - http://bmjopen.bmj.com/site/about/guidelines.xhtml

that TRs are in an ideal position to deliver advice on health behaviours, particularly when asked about nutrition advice delivery.

"They routinely see the specialist radiographer, for the breast patients. But they don't have a dietitian appointment" (TR 12).

"Prostate and breast are two tumour groups that are fully radiographer-led review and about 70% to 80% of our work load. They generally wouldn't be sent to a dietician" (TR 15).

"Only have a dietitian on board for the head and necks" (TR 9).

Discussion

TRs in this study saw themselves as well placed to deliver health behaviour advice, but also reported that they do not routinely provide advice to all patients. TRs were particularly unlikely to provide advice on healthy eating and physical activity, and were more likely to provide advice on those behaviours they believed would minimise radiotherapy or cancer related side effects. This is in line with previous research among TRs [15,28,37]. In one qualitative study a key facilitator reported among TRs in delivering smoking cessation support to patients was knowledge of the link between smoking and toxicity [28]. Another qualitative study that explored allied health professionals' views regarding the provision of dietary advice to patients, highlighted that TRs report giving dietary advice to help counteract the side effects of radiotherapy [37]. Additionally, in our study, if TRs did provide dietary advice, this tended to be general advice rather than cancer-specific advice on healthy eating.

In some studies, oncology HCPs have reported they do not self-identify as the right person to provide lifestyle advice [17,20]. However, in this study TRs identified themselves as being well placed to deliver health behaviour advice and in a unique position as a consistent member of the multidisciplinary team

For peer review only - http://bmjopen.bmj.com/site/about/guidelines.xhtml

providing care to patients. However, despite this, they do not feel qualified to deliver advice, particularly on the topic of healthy eating. In the UK, poor diet has the biggest impact on the NHS budget, greater than alcohol consumption, smoking and physical inactivity [38]. It has been noted that there are insufficient dietitians to provide dietary advice to all patients who may need dietary support [39]. In response to this all HCPs are being asked to implement a preventative healthcare approach within their role and the delivery of healthy eating advice is fundamental to this [23,24,40,41]. Key to achieving this is that TRs will have the skills, knowledge and behaviours to improve the health and wellbeing of individuals [24]. As with other oncology HCP groups [16,17,20,37] this study identifies the need for education and training among TRs in delivering health behaviour advice, particularly on healthy eating and physical activity. This training should also address when and how to refer to other support if necessary, as this was identified as a key facilitator in the delivery of advice on health behaviours, particularly when time is a barrier to the delivery of this advice [15,16,17].

All interviews demonstrated that TRs would welcome training on delivering health behaviour advice and recommended it as a key facilitator in delivering advice, in addition to incorporating it into the undergraduate setting. The need for postgraduate training among TRs in delivering health advice has also recently been reported by Charlesworth et al. [28] in relation to the delivery of smoking cessation advice. Our findings from this study provide additional insight into TRs preferences on the type of training on delivering lifestyle advice to those LWBC, with TRs demonstrating a preference of online training in the postgraduate setting. Amongst HCPs online education has been reported to be as effective as face-to-face education [42]. Additionally, the use of online learning enables HCPs to carry out training at time that fits in with clinical work [43, 44]. TRs in this study identified this benefit of online learning in overcoming the limited time available for TRs to undertake continuous professional development and additional training. Interestingly, TRs in this study mentioned having patient input in the training would be helpful. Whilst HCPs input is key to the development of interventions, patient members play key advocacy roles and their input can enhance the outcomes of interventions [45].

For peer review only - http://bmjopen.bmj.com/site/about/guidelines.xhtml

Patient input may also help overcome the reported barrier of fear of causing offence to a patient, which has been reported as a barrier amongst oncology HCPs in delivery of health behaviour advice [17].

Those LWBC wish to receive advice on health behaviours from their healthcare team [13,20,46] and is of particular importance as the period following a cancer diagnosis has been shown be a teachable moment and an ideal opportunity to motivate patients around the importance of healthy eating and physical activity [47,48]. This was made apparent in this study, whereby some TRs mentioned that healthy eating and exercise were the health behaviours patients ask for advice on more often, generally towards the end of their treatment. This further highlights the importance of supporting TRs in delivering evidence-based health behaviour advice to meet patients' needs.

TRs have a responsibility to educate patients on the importance of following healthy behaviours given the increasing evidence showing implementing healthy behaviours improve a number of physical and psychosocial outcomes after a cancer diagnosis [2,3]. Among pre- and post-menopausal women living with and beyond breast cancer, a systematic literature review and meta-analysis of 82 follow-up studies (n = 213075 breast cancer survivors) identified that being overweight increases the risk of all cause and breast cancer mortality [4]. Being physically active after a cancer diagnosis is also correlated with improved survival and reduced recurrence [5,6,49]. Whilst data is limited, emerging research suggests healthy dietary behaviours after a diagnosis may improve outcomes [3,50]. In a prospective observational study of 1009 patients with stage III colon cancer, a higher intake of a typical Western diet was associated with a three-fold increased risk of disease recurrence and a 2.3-fold increased risk of all-cause mortality [8]. Additionally, those LWBC are at increased risk for developing cardiovascular disease, osteoporosis, and diabetes and healthy behaviours can reduce the risk of developing these diseases [51,52].

For peer review only - http://bmjopen.bmj.com/site/about/guidelines.xhtml

Of those interviewed in this study it appeared that those with breast, prostate and colorectal cancer are primarily reviewed and assessed by TRs. Therefore, it is the responsibility of TRs to deliver advice on improving health behaviours to these patients. This is also particularly important because the strongest evidence for the benefits of diet and exercise is currently in breast, prostate and colorectal cancer survivors [53]. These are also the most common cancers in the UK and radiotherapy plays a key role in managing these cancers [22, 54]. Therefore, with the right skills and knowledge, TRs could deliver advice on improving health behaviours. By supporting self-efficacy among patients towards the end of their treatment which very often is in the radiotherapy department can be empowering for patients. Among those with prostate cancer, implementing dietary changes brought psychological benefit, as a method of coping and regaining control over their diagnosis [46].

Strengths and limitations

This is the first qualitative study among TRs to explore the provision of advice on all key modifiable lifestyle behaviours for those LWBC as per recommendations [2]. Whilst the aim of qualitative research is not to generalise the findings the sample size was small, and therefore the findings may not be representative of the views of the wider therapeutic radiography workforce. However, data saturation was reached, likely due the homogeneous sample of participants. Additionally, the participants worked in different radiotherapy departments and therefore provide insight into the practices among TRs in the delivery of healthy behaviour advice from a wide range of hospitals. Also, the participants worked in cancer centres in England, Wales, Scotland and Northern Ireland, providing insight into the practices across the UK. Another limitation of this study is the low response rate (20.8%) and that the participants might be more interested in the role of health behaviours in cancer survivorship, which might bias the responses towards a positive view on this topic and the role of TRs in delivering advice within their role. Despite this however, provision of health behaviour change advice was low, suggesting TRs may be even less likely to educate patients around the importance of healthy behaviours.

For peer review only - http://bmjopen.bmj.com/site/about/guidelines.xhtml

Future research

This study highlights the need for training and education among TRs on the delivery of health behaviour advice to cancer patients, both in the undergraduate and postgraduate setting. Particularly on the topics of physical activity, healthy eating and weight management. Higher Education Institutions have a responsibility in educating the Allied Health Professional workforce on implementing health promotion within their role [55]. Further research among pre-registration TR students and lecturers within therapeutic radiography should therefore explore how best to address this need. Future research among TRs should also use purposive sampling to identify the views and health promotion practices among those who may not have a primary interest in the area of health behaviours among those LWBC.

Conclusion

In conclusion, whilst the majority of TRs delivered some advice on health behaviours as part of their role, advice was mainly on smoking and alcohol intake. Most believed in the value of this advice in managing radiotherapy and cancer related side effects. Provision of advice was lowest for weight management, healthy eating and physical activity. The findings show a need for training among TRs in delivering advice on improving health behaviours among those LWBC, with TRs reporting a preference for online training in the post graduate setting.

Declarations

Contributors: NDP had the original idea for the study and obtained the funding with AF, RJB and KPJ. NDP developed the design of the study, acquired the data, analysed and interpreted the data, drafted and revised the article and approved the final the manuscript submitted. AF provided behavioural science expertise, contributed to the development of the study design, the recruitment approach,

For peer review only - http://bmjopen.bmj.com/site/about/guidelines.xhtml

analysed and interpreted the data, reviewed, edited and approved the final manuscript. RJB provided behavioural science expertise, contributed to the development of the study design, the recruitment approach, interpreted the data and reviewed, edited and approved the final manuscript. KPJ provided oncology expertise and intellectual input into the recruitment approach, design and approved the final manuscript. LC provided therapeutic radiography and public health expertise which informed the development of the study design and reviewed the article and approved the final manuscript. NW provided radiography and public health expertise and contributed to the development of the study design, reviewed the manuscript for important intellectual content and approved the final the manuscript submitted.

Competing Interests: None

Funding: This research was funded by Cancer Research UK (ref no: C59769/A25965).

Data sharing statement: Data can be obtained by the corresponding author on reasonable request.

Ethics approval: The methodology for this study was approved by the Human Research Ethics committee of University College London (reference 12945/001).

Consent to participate: Informed consent was obtained from all individual participants included in the study.

Acknowledgments: The researchers are grateful to the health professionals who participated in the study.

For peer review only - http://bmjopen.bmj.com/site/about/guidelines.xhtml

References

1. Brown KF, Rumgay H, Dunlop C, Ryan M, Quartly F, Cox A et al. The fraction of cancer attributable to modifiable risk factors in England, Wales, Scotland, Northern Ireland, and the United Kingdom in 2015. Br. J. Cancer. 2018;118(8):1130-41.

2. World Cancer Research Fund/American Institute for Cancer Research. Diet, Nutrition, Physical Activity and Cancer: a Global Perspective. Continuous Update Project Expert Report. 2018. [Internet] Available at: dietandcancerreport.org. Accessed 20 Jan 2020.

3. Thomas, R, Davies, N. Lifestyle during and after cancer treatment. Clin. Oncol. 2007;19(8): 616-627.

4. Chan DS, Vieira AR, Aune D, Bandera EV, Greenwood DC, McTiernan A et al. Body mass index and survival in women with breast cancer—systematic literature review and meta-analysis of 82 follow-up studies. Ann. Oncol. 2014 Oct 1;25(10):1901-14.

5. Speck, R.M., Courneya, K.S., Mâsse, L.C., Duval, S. & Schmitz, K.H. An update of controlled physical activity trials in cancer survivors: a systematic review and meta-analysis. J Cancer Surviv. 2010;4 (2):87-100.

6. Pierce JP, Stefanick ML, Flatt SW, Natarajan L, Sternfeld B, Madlensky L et al. Greater survival after breast cancer in physically active women with high vegetable-fruit intake regardless of obesity. J. Clin. Oncol. 2007;25(17):2345–2351.

7. World Cancer Research Fund/American Institute for Cancer Research. Continuous Update Project Expert Report. 2018. Survivors of breast and other cancers. [Internet] Available at: https://www.wcrf.org/sites/default/files/Breast-cancer-survivors-report.pdf. Accessed: 16 Jan 2020. Accessed 2 April 2020.

8. Meyerhardt JA, Niedzwiecki D, Hollis D, Saltz LB, Hu FB, Mayer RJ et al. Association of dietary patterns with cancer recurrence and survival in patients with stage III colon cancer. Jama. 2007;298(7):754-64.

9.Tollosa DN, Tavener M, Hure A, James EL. Adherence to multiple health behaviours in cancer survivors: a systematic review and meta-analysis. J Cancer Surviv. 2019;13(3):327-43.

For peer review only - http://bmjopen.bmj.com/site/about/guidelines.xhtml

10. Pugh G, Hough R, Gravestock H, Fisher A. The health behaviour status of teenage and young adult cancer patients and survivors in the United Kingdom. Support. Care Cancer. 2020;28(2):767-77

11. Corbett T, Cheetham T, Müller AM, Slodkowska-Barabasz J, Wilde L, Krusche A et al. Exploring cancer survivors' views of health behaviour change: "Where do you start, where do you stop with everything?". Psycho-oncology. 2018;27(7):1816-24.

12. Jones, L.W., Courneya, K.S., Fairey, A.S., Mackey, J.R. Effects of an oncologist's recommendation to exercise on self-reported exercise behavior in newly diagnosed breast cancer survivors: a single-blind, randomized controlled trial. Ann. Behav. Med. 2004;28(2):105-13

13. Beeken RJ, Williams K, Wardle J, Croker H. "What about diet?" A qualitative study of cancer survivors' views on diet and cancer and their sources of information. Eur. J. Cancer Care. 2016;25(5):774-83.

14. Daley AJ, Bowden SJ, Rea DW, Billingham L, Carmicheal AR. What advice are oncologists and surgeons in the United Kingdom giving to breast cancer patients about physical activity? Int. J. Behav. Nutr. Phys. Act. 2008;5(1):46.

15. Pattinson L, Jessop A. The delivery of health improvement information during radiotherapy treatment: a survey of UK therapy radiographers. J Radiother Pract. 2016; 15 (2): 114–130.

16. Williams K, Beeken RJ, Fisher A, Wardle J. Health professionals' provision of lifestyle advice in the oncology context in the United Kingdom. Eur J Cancer Care. 2015;24(4):522-30.

17. Koutoukidis DA, Lopes S, Fisher A, Williams K, Croker H, Beeken RJ. Lifestyle advice to cancer survivors: a qualitative study on the perspectives of health professionals. BMJ open. 2018;8(3).

18. Cantwell M, Walsh D, Furlong B, Moyna N, McCaffrey N, Boran L et al. Healthcare professionals' knowledge and practice of physical activity promotion in cancer care: Challenges and solutions. Eur J Cancer Care. 2018;27(2):12795.

19. Conlon K, Pattinson L, Hutton D. Attitudes of oncology healthcare practitioners towards smoking cessation: A systematic review of the facilitators, barriers and recommendations for delivery of advice and support to cancer patients. Radiography. 2017;23: 256-263

For peer review only - http://bmjopen.bmj.com/site/about/guidelines.xhtml

20. Sutton E, Hackshaw-McGeagh LE, Aning J, Bahl A, Koupparis A, Persad R et al. The provision of dietary and physical activity advice for men diagnosed with prostate cancer: a qualitative study of the experiences and views of health care professionals, patients and partners. Cancer Causes Control. 20171;28(4):319-29.

21. Spellman C, Craike M, Livingston PM. Knowledge, attitudes and practices of clinicians in promoting physical activity to prostate cancer survivors. Health Educ J. 2013:1– 10. 34.

22. Cancer Research UK. Vision for radiotherapy. 2014.

23. Halkett GK, Kristjanson LJ. Patients' perspectives on the role of radiation therapists. Patient Education and Counselling. 2007;69(1):76-83

24. Society and College of Radiographers (SCoR). The College of Radiographers Research Priorities for the Radiographic Profession. 2017

25. Chief Allied Heath Professions Officer's Team. Allied Health Professions into Action. Using Allied Health Professionals to transform health, care and wellbeing. 2017. NHS England

26. Allied Health Professions Federation. UK Allied Health Professions Public Health Strategic Framework 2019-2024. 2019. [Internet] Available at: http://www.ahpf.org.uk/files/UK%20AHP%20Public%20Health%20Strategic%20Framework%202019-2024.pdf. Accessed 17 Jan 2020.

27. NHS. An Implementation Guide and Toolkit for Making Every Contact Count: Using every opportunity to achieve health and wellbeing. 2016

28. Charlesworth L, Hutton D, Hussain H. Therapeutic Radiographers' perceptions of the barriers and enablers to effective smoking cessation support. Radiography. 2019;25(2):121-8.

29. Boaz, A, Baeza, J, Fraser, A. Effective implementation of research into practice: an overview of systematic reviews of the health literature. BMC research notes 2011; 4 (1): 212.

30. Gill P, Stewart K, Treasure E, and Chadwick B. Methods of data collection in qualitative research: interviews and focus groups. Br Dent J. 2008; 204(6):291-5.

For peer review only - http://bmjopen.bmj.com/site/about/guidelines.xhtml

31. Guest G, Bunce A, Johnson L. How many interviews are enough? An experiment with data saturation and variability. Field methods. 2006;18(1):59-82.

32. Saunders B, Sim J, Kingstone T, Baker S, Waterfield J, Bartlam B et al. Saturation in qualitative research: exploring its conceptualization and operationalization. Quality quantity. 2018;52(4):1893-907.

33. Gale NK, Heath G, Cameron E, Rashid S, Redwood S. Using the framework method for the analysis of qualitative data in multi-disciplinary health research. BMC medical research methodology. 2013;13(1):117.

34. Tong A, Sainsbury P, Craig J. Consolidated criteria for reporting qualitative research (COREQ): a 32-item checklist for interviews and focus groups. International journal for quality in health care. 2007;19(6):349-57.

35. Society of Radiographer. Radiotherapy radiographic workforce UK census 2018. [Internet] Available at https://www.sor.org/learning/document-library/radiotherapy-radiographic-workforce-uk-census-2018. Accessed 1 March 2020.

36. Williamson K, Williams KJ. Organisational justice, trust and perceptions of fairness in the implementation of agenda for change. Radiography. 2011;17(1):61-6.

37. McClinchy J, Williams J, Gordon L, Cairns M, Fairey G. Dietary Advice and Collaborative Working: Do Pharmacists and Allied Health Professionals Other Than Dietitians Have a Role? InHealthcare. 2015; 3(1):64-77. Multidisciplinary Digital Publishing Institute.

38. Scarborough P, Bhatnagar P, Wickramasinghe KK, Allender S, Foster C, Rayner M. The economic burden of ill health due to diet, physical inactivity, smoking, alcohol and obesity in the UK: an update to 2006–07 NHS costs. J. Public Health. 2011;33(4):527-35.

39. Thompson RL, Summerbell CD, Hooper L, Higgins JP, Little P, Talbot D et al. Dietary advice given by a dietitian versus other health professional or self-help resources to reduce blood cholesterol. Cochrane Database of Systematic Reviews. 2003(3).

40. The NHS. The NHS long term plan. 2019

For peer review only - http://bmjopen.bmj.com/site/about/guidelines.xhtml

41. Independent Cancer Taskforce. Achieving world-class cancer outcomes a strategy for England 2015-2020. 2015

42. Ruiz JG, Mintzer MJ, Leipzig RM. The impact of e-learning in medical education. Academic medicine. 2006; 81(3):207-12.

43. Childs S, Blenkinsopp E, Hall A, Walton G. Effective e-learning for health professionals and students—barriers and their solutions. A systematic review of the literature—findings from the HeXL project. Health Information & Libraries Journal. 2005;22:20-32.

44. Cook DA, Levinson AJ, Garside S, Dupras DM, Erwin PJ, Montori VM. Internet-based learning in the health professions: a meta-analysis. Jama. 2008;300(10):1181-96.

45. Gray-Burrows KA, Willis TA, Foy R, Rathfelder M, Bland P, Chin A, Hodgson S, Ibegbuna G, Prestwich G, Samuel K, Wood L. Role of patient and public involvement in implementation research: a consensus study. BMJ quality & safety. 2018;27(10):858-64.

46. Avery KN, Donovan JL, Horwood J, Neal DE, Hamdy FC, Parker C et al. The importance of dietary change for men diagnosed with and at risk of prostate cancer: a multi-centre interview study with men, their partners and health professionals. BMC family practice. 2014;15(1):81.

47. Denmark et al. 2000) Demark-Wahnefried W, Peterson B, McBride C, Lipkus I, Clipp E. Current health behaviors and readiness to pursue life-style changes among men and women diagnosed with early stage prostate and breast carcinomas. Cancer. 2000;88(3):674–84

48. Murphy, J.L. & Girot, E.A. The importance of nutrition, diet and lifestyle advice for cancer survivors–the role of nursing staff and interprofessional workers. J Clin Nurs. 2013;22(11-12):1539-49.

49. Lahart, I.M., Metsios, G.S., Nevill, A.M., Carmichael, A.R. Physical activity, risk of death and recurrence in breast cancer survivors: A systematic review and meta-analysis of epidemiological studies. Acta Oncol. 2015;54(5):635-54.

50. Van Blarigan EL, Meyerhardt JA. Role of physical activity and diet after colorectal cancer diagnosis. Journal of Clinical Oncology. 2015 Jun 1;33(16):1825.

For peer review only - http://bmjopen.bmj.com/site/about/guidelines.xhtml

51. Macmillan Cancer Support. (2015) The burden of cancer and other long-term health conditions. [Internet] Available at: https://www.macmillan.org.uk/documents/press/cancerandotherlong-termconditions.pdf. Accessed 27th Match 2020.

52. Cummings A, Grimmett C, Calman L, Patel M, Permyakova NV, Winter J, et al. Comorbidities are associated with poorer quality of life and functioning and worse symptoms in the 5 years following colorectal cancer surgery: results from the ColoREctal well-being (CREW) cohort study. Psychooncology. 2018;27(10):2427–35

53. Marmot M, Atinmo T, Byers T, Chen J, Hirohata T, Jackson A et al. Food, nutrition, physical activity, and the prevention of cancer: a global perspective. Office for national statistics. 2019. Cancer registration statistics, England: 2017.

54. National Cancer Registration & Analysis Service and Cancer Research UK: Chemotherapy, Radiotherapy and Tumour Resections in England: 2013-2014 workbook. London: NCRAS; 2017.

55. Council of Deans of Health. (2017). Guidance: Public Health Content within the Pre-Registration Curricula for Allied Health Professions. [Internet] Available at: https://councilofdeans.org.uk/wp-content/uploads/2017/09/Public-Health-Content-within-the-Pre-Registration-Curricula-for-Allied-Health-Professions.pdf. Accessed 20 January 2020.

For peer review only - http://bmjopen.bmj.com/site/about/guidelines.xhtml

Supplementary material 1: Interview topic guide

Topic	Question	Prompt
**Introduction**	Brief introductions; get verbal consent to tape-record the interview; remind the aims of study, check length of interview/audio-recorded.	
**Health promotion beliefs**	What are your views on the role of health behaviours in those with a diagnosis of cancer?<br><br>Do you think delivering advice on improving health behaviours is part of your role?	Breast/prostate/colorectal/head & neck cancers<br><br>Healthy eating/ exercise/ alcohol/ smoking / weight management
**Provision of advice on healthy behaviours**	Can you tell me about your experiences of providing health behaviour advice to cancer patients?<br><br>Are there some health behaviours you recommend more than others?<br><br>Why do you think that is?<br><br>Are there some patient groups that you provide health behaviour advice to more than others?<br><br>Why do you think that is?	Healthy eating/ exercise/ alcohol/ smoking / weight management
**Barriers to health advice delivery**	On the survey we asked you about a number of barriers limiting you in providing health behaviour advice to your patients. You mentioned X Y Z<br><br>Can you tell me more about these barriers?<br><br>Can you think of any other barriers within your role and work environment stopping you from giving lifestyle and health promotion advice to patients?	Why do you think that is?
**Addressing barriers**	What support do you think would be most helpful in enabling therapeutic radiographers to deliver health behaviour advice to patients?	
**Training preferences**	How helpful do you think an online training course specifically for therapeutic radiographers on delivering health behaviour advice to cancer patients would be?<br><br>How helpful do you think a face to face training course specifically for therapeutic radiographers on delivering health behaviour advice to cancer patients would be?<br><br>If you were provided with a training course (online or face to face) what topics would you like delivered?	Why do you think this is?
**Finish**	Thank you for your time. Do you have anything else you would like to add?	

For peer review only

COREQ (COnsolidated criteria for REporting Qualitative research) Checklist

A checklist of items that should be included in reports of qualitative research. You must report the page number in your manuscript where you consider each of the items listed in this checklist. If you have not included this information, either revise your manuscript accordingly before submitting or note N/A.

Topic	Item No.	Guide Questions/Description	Reported on Page No.
**Domain 1: Research team and reflexivity**			
Personal characteristics			
Interviewer/facilitator	1	Which author/s conducted the interview or focus group?	
Credentials	2	What were the researcher's credentials? E.g. PhD, MD	
Occupation	3	What was their occupation at the time of the study?	
Gender	4	Was the researcher male or female?	
Experience and training	5	What experience or training did the researcher have?	
Relationship with participants			
Relationship established	6	Was a relationship established prior to study commencement?	
Participant knowledge of the interviewer	7	What did the participants know about the researcher? e.g. personal goals, reasons for doing the research	
Interviewer characteristics	8	What characteristics were reported about the inter viewer/facilitator? e.g. Bias, assumptions, reasons and interests in the research topic	
**Domain 2: Study design**			
Theoretical framework			
Methodological orientation and Theory	9	What methodological orientation was stated to underpin the study? e.g. grounded theory, discourse analysis, ethnography, phenomenology, content analysis	
Participant selection			
Sampling	10	How were participants selected? e.g. purposive, convenience, consecutive, snowball	
Method of approach	11	How were participants approached? e.g. face-to-face, telephone, mail, email	
Sample size	12	How many participants were in the study?	
Non-participation	13	How many people refused to participate or dropped out? Reasons?	
Setting			
Setting of data collection	14	Where was the data collected? e.g. home, clinic, workplace	
Presence of non-participants	15	Was anyone else present besides the participants and researchers?	
Description of sample	16	What are the important characteristics of the sample? e.g. demographic data, date	
Data collection			
Interview guide	17	Were questions, prompts, guides provided by the authors? Was it pilot tested?	
Repeat interviews	18	Were repeat inter views carried out? If yes, how many?	
Audio/visual recording	19	Did the research use audio or visual recording to collect the data?	
Field notes	20	Were field notes made during and/or after the inter view or focus group?	
Duration	21	What was the duration of the inter views or focus group?	
Data saturation	22	Was data saturation discussed?	
Transcripts returned	23	Were transcripts returned to participants for comment and/or	

For peer review only

Topic	Item No.	Guide Questions/Description	Reported on Page No.
		correction?	
**Domain 3: analysis and findings**			
Data analysis			
Number of data coders	24	How many data coders coded the data?	
Description of the coding tree	25	Did authors provide a description of the coding tree?	
Derivation of themes	26	Were themes identified in advance or derived from the data?	
Software	27	What software, if applicable, was used to manage the data?	
Participant checking	28	Did participants provide feedback on the findings?	
Reporting			
Quotations presented	29	Were participant quotations presented to illustrate the themes/findings? Was each quotation identified? e.g. participant number	
Data and findings consistent	30	Was there consistency between the data presented and the findings?	
Clarity of major themes	31	Were major themes clearly presented in the findings?	
Clarity of minor themes	32	Is there a description of diverse cases or discussion of minor themes?	

Developed from: Tong A, Sainsbury P, Craig J. Consolidated criteria for reporting qualitative research (COREQ): a 32-item checklist for interviews and focus groups. *International Journal for Quality in Health Care*. 2007. Volume 19, Number 6: pp. 349 – 357

Once you have completed this checklist, please save a copy and upload it as part of your submission. DO NOT include this checklist as part of the main manuscript document. It must be uploaded as a separate file.

For peer review only
